# Focused ultrasound on the substantia nigra enables safe neurotensin-polyplex nanoparticle-mediated gene delivery to dopaminergic neurons intranasally and by blood circulation

**DOI:** 10.1186/s11671-024-04005-9

**Published:** 2024-04-02

**Authors:** Juan U. Mascotte-Cruz, Arturo Vera, Lorenzo Leija, Francisco E. Lopez-Salas, Michael Gradzielski, Joachim Koetz, Bismark Gatica-García, C. P. Rodríguez-Oviedo, Irais E. Valenzuela-Arzeta, Lourdes Escobedo, David Reyes-Corona, ME. Gutierrez-Castillo, Minerva Maldonado-Berny, Armando J. Espadas-Alvarez, Carlos E. Orozco-Barrios, Daniel Martinez-Fong

**Affiliations:** 1 Departamento de Fisiología, Biofísica y Neurociencias, Centro de Investigación y de Estudios Avanzados, Av. Instituto Politécnico Nacional No. 2508, San Pedro Zacatenco, 07360 Ciudad de México, México; 2 Departamento de Ingeniería Eléctrica-Bioelectrónica, Centro de Investigación y de Estudios Avanzados, Ciudad de Mexico, México; 3 Departamento de Biología Molecular y Biotecnología, Instituto de Investigaciones Biomédicas, Universidad Nacional Autónoma de México Instituto de Investigaciones Biomédicas, Ciudad de Mexico, México; 4 Institut für Chemie, Stranski-Laboratorium für Physikalische und Theoretische Chemie, Technische Universität Berlin, Berlin, Germany; 5 Institut für Chemie , Universität Potsdam, Potsdam, Germany; 6 Nanoparticle Therapy Institute, Aguascalientes, México; 7 Centro Interdisciplinario de Investigaciones y Estudios Sobre Medio Ambiente y Desarrollo, Departamento de Biociencias e Ingeniería, Instituto Politécnico Nacional, Ciudad de Mexico, México; 8 CONAHCYT - Unidad de Investigaciones Médicas en Enfermedades Neurológicas, Hospital de Especialidades “Dr. Bernardo Sepúlveda”, Centro Médico Nacional Siglo XXI, Instituto Mexicano del Seguro Social, Ciudad de Mexico, México

**Keywords:** Transient neuroinflammation, Reversible neurodegeneration, Gene transfection, Motor behavior, Nanomedicine, Bionanotechnology, Parkinson’s disease

## Abstract

Neurotensin-polyplex nanoparticles provide efficient gene transfection of nigral dopaminergic neurons when intracerebrally injected in preclinical trials of Parkinson’s disease because they do not cross the blood–brain barrier (BBB). Therefore, this study aimed to open BBB with focused ultrasound (FUS) on the substantia nigra to attain systemic and intranasal transfections and evaluate its detrimental effect in rats. Systemically injected Evans Blue showed that a two-pulse FUS opened the nigral BBB. Accordingly, 35 μL of neurotensin-polyplex nanoparticles encompassing the green fluorescent protein plasmid (79.6 nm mean size and + 1.3 mV Zeta-potential) caused its expression in tyrosine hydroxylase(+) cells (dopaminergic neurons) of both substantiae nigrae upon delivery via internal carotid artery, retro-orbital venous sinus, or nasal mucosa 30 min after FUS. The intracarotid delivery yielded the highest transgene expression, followed by intranasal and venous administration. However, FUS caused neuroinflammation displayed by infiltrated lymphocytes (positive to cluster of differentiation 45), activated microglia (positive to ionized calcium-binding adaptor molecule 1), neurotoxic A1 astrocytes (positive to glial fibrillary acidic protein and complement component 3), and neurotrophic A2 astrocytes (positive to glial fibrillary acidic protein and S100 calcium-binding protein A10), that ended 15 days after FUS. Dopaminergic neurons and axonal projections decreased but recuperated basal values on day 15 after transfection, correlating with a decrease and recovery of locomotor behavior. In conclusion, FUS caused transient neuroinflammation and reversible neuronal affection but allowed systemic and intranasal transfection of dopaminergic neurons in both substantiae nigrae. Therefore, FUS could advance neurotensin-polyplex nanotechnology to clinical trials for Parkinson’s disease.

**Supplementary Information:**

The online version contains supplementary material available at 10.1186/s11671-024-04005-9.

## Introduction

Neurotensin (NTS)-polyplex nanoparticles (NPs) constitute a specific gene delivery system to dopaminergic neurons that holds great promise for the therapy of Parkinson’s disease (PD) [1]. Those NPs result from the compaction of a plasmid DNA (pDNA) that contains the coding sequence of a neurotrophic factor gene under the control of dopamine transporter gene promoter (DAT) [2, 3] or the synthetic NBRE3x promoter [4–6], both specific for dopaminergic neurons, or an eukaryotic promoter such as elongation factor α1 promoter [7]. pDNA compaction is mainly carried out by the electrostatic binding of linear poly-L-lysine conjugated with NTS and a fusogenic peptide [8]. NTS-polyplex NPs are internalized by neurotensin receptor type 1 (NTSR1)-mediated endocytosis in dopaminergic neurons known to possess the highest density of membranal NTSR1 in the brain [1, 8, 9]. The fusogenic peptide translocates the NPs to the cytoplasm, thus avoiding pDNA endosomal degradation [3, 8]. A karyophilic peptide, also electrostatically bound to the pDNA, contributes to pDNA compaction and targets it to the cell nucleus through the α/β importin system to express the codifying sequence [10]. Physical characterization of NTS-polyplex NPs using transmission and scanning electron microscopies and dynamic light scattering shows that their size ranges from 50 to 150 nm and possess an electrical surface charge of 22 ± 7 mV [3, 5, 7, 11, 12]. These physical characteristics likely prevent intravenously injected NTS-polyplex NPs from crossing the blood–brain barrier (BBB) [12, 13]. In addition, NTS-polyplex NP physical features have been the basis for establishing their pharmaceutical profile for clinical use [11]. Those NPs remain stable in bovine and human serum after 60 min incubation, their peptidic part degradation products are cleared mainly by urine, and lyophilized NPs remain functional for at least six months of storage at 25 °C and 60% relative humidity [11]. The safety of NTS-polyplex NPs is a competitive advantage for human use because they do not activate systemic and local innate immune responses [14]; on the contrary, they produce an anti-neuroinflammatory effect when used in therapeutic gene delivery [6, 7]. Specificity is another advantage of NTS-polyplex NPs provided by the NTS ligand, which internalizes them through endocytosis of NTSR1, highly expressed in dopaminergic neurons [2, 8, 9, 15, 16]. The ligand-mediated specificity can be additionally reinforced with a tissue-specific promoter for dopaminergic neurons, such as DAT or NBRE3x promoters [4, 6, 16, 17]; furthermore, an inducible doxycycline-regulated system can control the transgene expression if needed [5]. Moreover, the ability of dual transfection with unilateral administration is another advantage [7]. Those features make NTS-polyplex attractive for clinical use in PD.

PD is a neurodegenerative ailment caused by the progressive loss of dopaminergic neurons and with existing symptomatic but no curative treatment [1]. Because of the limitations of dopaminergic replacement therapy, based on the drug [18] or gene delivery [19], neurotrophic factor therapy via protein local injections or gene delivery has emerged with the potential to stop neurodegeneration and restore the dopaminergic nigrostriatal system. Cerebral dopamine neurotrophic factor (CDNF) is an example of a novel therapy that can be used by injecting the CDNF protein or by CDNF gene delivery in preclinical PD models [20]. Other neurotrophic factors, such as glial-cell line-derived neurotrophic factor (GDNF) and neurturin (NRTN), are currently evaluated in clinical trials by purified protein injections or viral vector-mediated gene delivery to the brain [21]. To date, those therapies have been applied through a local injection in the brain or infusion in the lateral cerebral ventricle. The therapeutic potential of NTS-polyplex NPs relies on their ability to deliver genetic cargos via NTSR1-mediated internalization to dopaminergic neurons in vitro and in vivo [8, 16]. Preclinical studies with 6-hydroxydopamine (6-OHDA)-induced early parkinsonism have shown that the targeted delivery of genes coding for neurotrophic factors such as human (hGDNF) [22], rat brain-derived neurotrophic factor (rBDNF) [2], or hCDNF [6] can rescue dopaminergic neurons from neurodegeneration. Furthermore, gene delivery of hCDNF, rBDNF, or mNRTN in rats with 6-OHDA-induced advanced parkinsonism restores the structure and function of the dopaminergic nigrostriatal system [4, 17, 23]. A recent study in a novel PD model shows that the NTS-polyplex NP-mediated *nurr1* gene transfection in dopaminergic neurons significantly reduces neuroinflammation, prion-like spreading of pathological α-synuclein aggregates, and degeneration of the dopaminergic nigrostriatal system, thus improving locomotor activity [7]. However, invasive intracerebral injection limits NTS-polyplex NPs for clinical translation because they do not permeate the blood–brain barrier (BBB) when administered via blood circulation [13].

Recent research has demonstrated that focused ultrasound (FUS) is a minimally invasive technique that can open the BBB locally and reversibly to allow therapeutics to cross to the brain parenchyma after intravenous administration [24]. The exact cellular and molecular mechanisms of BBB permeation by FUS treatment are under investigation, although it has been suggested that shear stress on the BBB triggers transcellular and paracellular transport [25]. Regardless, FUS has been successfully used in the targeted delivery of specificity-lacking genetic material to the desired brain region after sanguineous administration in wild-type rodents, such as a luciferase-coding plasmid using brain-penetrating NPs [26], luciferase mRNA encapsulated in lipid NPs [27], or designer receptors exclusively activated by designer drugs (DREADDs) delivered by adeno-associated virus (AAV) serotype 9 vector [28]. FUS has also managed to deliver neurotrophic factors, whether proteins or genes, to specific brain regions systemically transported by different vehicles in healthy rodents; for instance, BDNF plasmid encapsulated in cationic bubbles [29], and pBDNF-EGFP-loaded microbubbles [30], or NRTN alone [31, 32]. Notably, FUS has increased the intranasal delivery to rodent brains of BDNF protein [33] and hGDNF plasmid compacted with polyethylene glycol [34]-polylysine [34]. From the therapeutic point of view, FUS has enabled access to the brain of the proteins BDNF intranasally delivered [35] and NRTN intravenously injected [32] in neurotoxin-induced PD animal models. FUS has also yielded similar therapeutic effects by GDNF gene delivery through AAV [32], liposomes [36, 37], cationic microbubbles [38], and polyethyleneimine-polyethyleneglycol NPs [39]. Nrf2 transfection using FUS and MRI-guided delivery of nano-microbubbles in rats also protects dopaminergic neurons from the 6-OHDA lesion [40]. These pioneer studies have focused on demonstrating the proof-of-principle of FUS-based neurotrophic factor therapy, either as protein or gene; however, none has addressed if such an approach may activate neuroinflammation in the substantia nigra, where dopaminergic neurons dwell. Therefore, It is crucial to investigate whether FUS can elicit neuroinflammation and its impact on dopaminergic neurons, especially considering that neuroinflammation significantly contributes to PD pathology through microglia-triggered neurotoxic A1 astrocytes, which are lethal for neurons and oligodendrocytes [41, 42].

The transiently increased temperature in the brain is the primary concern of FUS intended to open the BBB in the substantia nigra [43]. We have previously reported that a two-pulse FUS protocol (3 watts, 1.965 MHz, for 2 min with 30 s of intermittence) caused a 4 °C temperature increase in the focus area for 2.5 min during ultrasound exposure [44]. Although this increment is well tolerated in other brain regions [45, 46], it may be harmful in the substantia nigra because dopaminergic neurons are highly vulnerable to internal and external insults [47]. One of the most known internal insults is dopamine nonenzymatic oxidation products, which are toxic to dopaminergic neurons through different mechanisms [48]. In addition, the insufficient antioxidant system response caused by naturally reduced total glutathione levels in nigral dopaminergic neurons [49] and the high density of the microglia population in the healthy substantia nigra [50] are other internal factors that can favor neuroinflammation. Concerning gene delivery, the gene carrier can be considered an external insult because its structure and amount could cause physical damage, and its components acting as an antigen could trigger neuroinflammation. These critical issues remain unclear and should be investigated to propose the true scope of NTS-polyplex NPs in PD gene therapy.

Since NTS-polyplex NPs do not cross the BBB [12, 13], the first objective of this work was to implement their delivery via the nasal mucosa and blood circulation to dopaminergic neurons by applying FUS on the substantia nigra[44]. Because NTS-polyplex NPs are safe [3, 6, 14], the second objective was to determine whether FUS-facilitated transfection could aggravate neuroinflammation known to occur in FUS [51], inducing neurotoxic A1 astrocyte appearance and neuronal damage in the substantia nigra. Evans Blue (EB) dye was used as a positive control of BBB opening [52]. Immediately after FUS, the same amount of NTS-polyplex NPs harboring the enhanced green fluorescent protein (GFP) plasmid, proven safety in vitro [3, 10] and in vivo [14], was delivered via the internal carotid artery, retro-orbital venous sinus, or nasal mucosa [52] to demonstrate GFP expression in nigral dopaminergic neurons and axonal projections. The harmful effect of this procedure was evaluated through neuroinflammation cell variables and the condition of the dopaminergic nigrostriatal system over time. This study shows for the first time that FUS overcame BBB limitation for systemic transfections of NTS-polyplex NPs, opening three avenues for their delivery: intracarotid, intravenous, and intranasal. Of interest for future clinical uses, intracarotid administration was the most efficient and safe due to the induction of neurotrophic A2 astrocytes, which possibly overcame the lethal effect of neurotoxic A1 astrocytes on dopaminergic neurons; additionally, it maintained the specificity and dual transfection ability of NTS-polyplex NPs. Our FUS protocol and contribution to neuroinflammation knowledge can also benefit other gene vectors.

## Experimental part

### Plasmid

pEGFP-N1 (4733 bp) codes for enhanced GFP under the control of the cytomegalovirus promoter (Clontech Laboratories, Inc; East Meadow Circle, Palo Alto, CA, USA).

### NTS-polyplex NP assembling

NTS-polyplex NPs result from the compaction of a plasmid DNA by a karyophilic peptide (KP) and the NTS carrier in two successive electrostatic biding steps widely described elsewhere [3, 8]. To select the suitable NPs for systemic and intranasal gene delivery, NTS-polyplex NPs were assembled with the plasmid pEGFP-N1, the NTS carrier, and the KPRa (Ac-KMAPKKRK) or KPSV40 (Ac-MAPTKRKGSCPGAAPNKPK) or without these peptides in Dulbecco’s Modified Eagle Medium (DMEM; Thermo Fisher Scientific-MX, Ciudad de México, MX) at an optimum molar ratio. First, a KP (9 μM) was electrostatically bound to the plasmid pEGFP-N1 (18 nM) to form the plasmid-KP complex. Second, this complex was compacted into NPs by electrostatically attaching the NTS carrier (432 nM), which was previously synthesized by cross-linking NTS and HA2 fusogenic peptide (PF) with poly-L-lysine [3, 8]. NTS-polyplex NPs were also assembled without a KP as a control of physical characterization. Therefore, the total pDNA was 1.69 µg in 35 μL of DMEM per rat and was similar for transfections via the internal carotid artery, retro-orbital sinus, and nasal mucosa.

### Cryo-scanning electron microscopy

The form and size of NTS-polyplex NPs were examined using a high-resolution Cryo-Scanning Electron Microscope (Cryo-SEM; Hitachi S-4800; Tokyo, Japan). The samples were frozen in melting nitrogen and fractured in the cryo chamber at − 120 °C. After the etching process at -98 °C for 45 s, the samples were sputtered for 120 s with platinum in the GATAN Alto 2500 Cryo-preparation chamber and transferred into the Cryo-SEM. Pictures were taken with objectives 20X and 80X.

### Dynamic light scattering

The hydrodynamic diameter and the surface charge of NTS-polyplex NPs were determined by dynamic light scattering (DLS) using a Litesizer™ 500 apparatus equipped with a 658 nm laser (Anton Paar; Graz, Austria). The size was measured in 700 µL freshly prepared samples in pre-rinsed polystyrene cuvettes using a backscatter angle of 175°. Each measurement included 60 runs with 10 s intervals at 25 °C; the optical filter density and the focus were automatically selected. In addition, the surface charge of NTS-polyplex NPs was calculated through zeta-potential measurements using electrophoretic light scattering (ELS). The measurements were made in 300 µL of fresh samples in omega cuvettes containing gold electrodes. The Litesizer™ 500 apparatus was set to 60 runs at 200 V and 25 °C.

### Size measurement with Fiji of NTS-polyplex

The images taken by Cryo-SEM were converted to binary and processed using ImageJ software [53]. Initially, the scale size is designated using the straight command, which indicates the calibration scale of the image, and this is processed with the set scale command to obtain a value of pixels/nm. After this procedure, a threshold adjustment is made to delimit the areas of each NP. The straight tool was used to delimit the length of each NP and then proceed with the measurement to obtain the values [53].

### Animals

All the animal work was conducted according to protocol #162–15 protocol (authorization No. 162–15; approval date: June 9, 2019), authorized and supervised by the Institutional Committee for the Care and Use of Laboratory Animals of the Center for Research and Advanced Studies (CINVESTAV) following the current Mexican legislation, NOM-062-ZOO-1999, and NOM-087-ECOL-1995 (Secretaría de Agricultura, Ganadería, Desarrollo Rural, Pesca y Alimentación; SAGARPA). Male Wistar rats weighing between 210 and 230 g were supplied by the Animal Production and Experimentation Unit of CINVESTAV and maintained at 22 °C, 60 ± 5% humidity, with access to food and water ad libitum. A total of 66 animals were used, which was the minimum number according to the experimental design and statistical validity in compliance with the Guide for the Care and Use of Laboratory Animals (The National Academies Collection: Reports funded by National Institutes of Health, 2011) and considering the three R’s (Reduction, Refinement, and Replacement) for animal experimentation [54]. Moreover, all efforts were made to avoid animal suffering. Surgical procedures were performed under general anesthesia using a mixture of xylazine and ketamine (9 mg and 120 mg/kg; PISA Laboratories; Mexico City, MX). Animals (*n* = 66 in total) were randomly separated into two groups with subgroups each: (1) FUS + EB, 18 rats for EB dye administration after FUS distributed according to the administration route: internal carotid artery, 3 rats; retro-orbital venous sinus, 3 rats; caudal vein, 3 rats; nasal mucose, 3 rats, observation in 300 µm-thick fresh mesencephalon, 3 rats. Additionally, 3 rats injected with EB via caudal vein without FUS formed the negative control group. (2) FUS + Transfection, 48 rats transfected with pEGFP-N1 plasmid after FUS application. Twelve rats were used for GFP expression after delivery by internal carotid artery (3 rats), nasal mucosa (3 rats), and retro-orbital venous sinus (3 rats). Additionally, 3 rats transfected by the internal carotid artery were used for immunofluorescence staining controls. For studies of the time course of neuroinflammation, neuronal damage, and behavior after transfection by internal carotid artery, 36 rats were used distributed in the following groups: 1 day (6 rats), 7 days (6 rats), 15 days (6 rats), 60 days (6 rats). CTRL (6 untransfected rats without FUS), SHAM (6 rats with craniotomy without FUS). (Supplementary file 1: Fig. S1).

### Craniotomy and FUS application

Anesthetized rats were fixed with a stereotaxic device (Stoelting; Wood Dale, ILL, USA), their skulls were cleaned with hydrogen peroxide, and a 1 cm2 square craniotomy was performed in the left side of the skull after dissecting the head tissues. The corresponding bone piece was removed after making the square cut with a mini drill (Dremel®, Multipro™, model 395) and drill bit of 1 mm to place the FUS transducer cone over the dura mater at the coordinates of + 2 mm on the transverse axis and + 2.1 mm on the anteroposterior axis, taking the lambda point as a reference. An ultrasonic gel was applied between the cone and the exposed brain area to avoid gas bubbles and allow the most efficient transmission of the ultrasonic waves. The skull bone piece, conserved in a cold, sterile Hartmann solution, was repositioned and attached with bone wax (Atramat; Mexico City, MX) after FUS completion, and the skin was sutured.

FUS was applied through a cone (thermoplastic polyurethane, TPU) filled with water by using a 2 MHz (nominal frequency) monoelemental concave transducer (Onda Corporation; Sunnyvale, CA, USA) with a focal length of 20 mm and a radius of 10 mm. An acoustic gel was used as an intermediate coupler between the cone and the rat head to transmit ultrasound efficiently. The FUS transducer was driven by a signal generator (2052, Teledyne LeCroy; New York, USA) and a power amplifier (500A250, Amplifier Research; Souderton, PA, USA), providing a sine wave of 1,965 MHz (work frequency) modulated with a tone burst of 10 Hz. The transducer was monitored by a power meter (PM2002, Amplifier Research, Gainesville, GA, USA). An oscilloscope (600A, LeCroy; Lake Mary, USA) was used to monitor the output signal. The signal strength was applied following the protocol established previously [44] for the temporary opening of the BBB in the substantia nigra (Supplementary file 1: Fig. S2).

### Catheter design and dissection of the right internal carotid artery

The internal carotid artery of Wistar rats has an external diameter of 0.95 mm and an internal diameter of 0.71 mm [55]. Therefore, suitable 15 cm long catheters were made of STT-28 PTFE tube with 0.38 mm inner diameter (Light Wall; Sparta, TN, USA) by stretching the ends with the help of forceps while heating the middle part with a Bunsen burner. Next, a diagonal cut was made with a stainless-steel blade in the thinned region to obtain an acute angle of 0.38 ± 5 mm thickness. Finally, the catheter tip was quickly passed over the lighter flame to remove imperfections from cutting [56].

The right internal carotid artery was exposed in anesthetized rats. First, a 1 cm incision was made in the middle line of the neck. Then, fatty tissue and muscle around the trachea were dissected to expose the sternocleidohyoid muscle using the submandibular gland as a reference since the internal carotid artery runs parallel together with the nerve vagus and the internal jugular vein within the neurovascular bundle. Once the internal carotid artery was identified through its steady beat, the vagus nerve was separated using beak forceps, and the catheter was inserted into the internal carotid artery. After infusing the adequate volume of the testing solutions by a microinfusion pump (10 µL/min), the internal carotid artery was occluded for 5 s at both ends to remove the catheter and seal the wound with electrocautery (Weller; GS Deventer, NL). Upon confirming correct cauterization, the occlusion clamps were removed, the surgical planes were sutured, and the rat was left under a heat source until complete recovery.

### Evans blue dye and NTS-polyplex NPs with the GFP plasmid

A 2% EB dye (960.81 Da; Sigma-Aldrich; Saint Louis, MO, USA) solution in phosphate-buffered saline (PBS) was used to evaluate the transient BBB opening because its adherence to serum albumin avoids crossing the BBB and its safety in short-term evaluations via blood circulation [57]. In addition, different volumes of EB were used to select the route that supplies a higher concentration in the substantia nigra with a minor injection volume to be utilized for NTS-polyplex NP administration. The injected volumes agreed with the Refining Procedures for The Administration of Substances through the caudal vein, retro-orbital venous sinus, internal carotid artery, and intranasally [58]. At 30 min after FUS, EB solution was injected for 3 min in a bolus of 300 μL via the caudal vein, 150 μL via the right retro-orbital venous sinus [44], 100 μL via intranasal [52], and 75 μL via the internal carotid artery [58]. Thirty minutes after the EB injection, the rats were perfused to obtain 30-μm brain cuts, as described below. Thirty-five μL of NTS-polyplex NPs harboring the pEGFP-N1 plasmid were injected through a microperfusion pump (Mod. 100; Stoelting; Wood Dale, IL, USA) at a 10 µL/min flow rate equipped with a 50 μL Hamilton syringe. Two weeks after administration, double immunofluorescence against TH and GFP was used to show GFP expression in dopaminergic neurons and axonal projections. In all injections, 5 μL of 10 U/mL heparin solution (Laboratorios Pisa; Guadalajara, JAL, MX) was filled in the catheter tip after loading the EB solution or NTS-polyplex NPs to avoid coagulation.

#### Brain dissection

The brains were immediately removed from rats, anesthetized with pentobarbital (50 mg/Kg, i.p.), and cut into 300 μm coronal sections with a vibratome at 4 °C (Leica VT 1200; Heidelberg, GER) for a quick detection of EB. For a detailed EB location, another rat lot was anesthetized with pentobarbital (50 mg/Kg, i.p.) and perfused through the ascending aorta with 100 ml of cold PBS, followed by 100 mL of 4% paraformaldehyde in PBS the following day after EB administration. Then, the brain was cut into 30-µm coronal sections using a sliding microtome at − 18 °C (Leica SM2010R; Heidelberg, GER).

For immunostaining assays, the brains were removed and kept in paraformaldehyde for 24 h at 4 °C and then cryoprotected in a PBS containing 30% sucrose at 4 °C overnight. The brain was cut into 30 µm thick serial coronal sections at the level of the substantia nigra and striatum using a sliding microtome at − 18 °C (Leica SM2010R; Heidelberg, GER). The sections were consecutively collected in a 24-well plate containing PBS, washed with PBS for 5 min, permeabilized with 0.1% Triton in PBS for 20 min, and incubated with albumin 1% bovine serum (BSA) in 0.1% Triton in PBS for 30 min to block nonspecific binding sites. After incubation in each procedure step described above, the samples were washed thrice with PBS for 5 min. Finally, the slices were used for immunohistochemistry and immunofluorescence staining.

#### Immunofluorescence

Activated microglia, astrogliosis, and CD45 cells were assessed using double immunofluorescence with dopaminergic neurons to localize those cells in the substantia nigra. The astrocyte type was identified by double immunofluorescence against specific epitopes using a proper combination of primary and secondary antibodies (Supplementary file 1: Table S1). Slices were incubated overnight at 4 °C with one of the following primary antibodies: mouse monoclonal anti-TH (1:1000; Sigma Aldrich; St. Louis, MO, USA), rabbit polyclonal anti-TH (1:1000; Millipore; Temecula, CA, USA), goat polyclonal anti-Iba1 (1:500; Abcam; Cambridge, UK), mouse monoclonal anti-GFAP (1:500; Clone GA5; Cell Signaling Technology; Danvers, MA, USA), mouse anti-CD45 (1:50; BD Bioscience; Sparks Glencoe, MD, USA), rabbit polyclonal anti-S100 calcium-binding protein A10 (S100A10; 1:100; Invitrogen, Carlsbad, CA, USA), rabbit polyclonal anti-C3 (1:100; Abcam; Cambridge, UK) and rabbit polyclonal anti-GFAP (1:1000; Abcam; Cambridge, UK). Finally, the slices were incubated for 2 h at room temperature (RT) with the suitable secondary antibody among Alexa Fluor 488 anti-mouse H+L chicken IgG (1:300; Invitrogen Molecular Probes; Eugene, OR, USA), Alexa Fluor 488 anti-rabbit H+L chicken IgG (1:300; Invitrogen Molecular Probes; Eugene, Oregon), Alexa Fluor 555 donkey anti-goat H+L IgG (1:300; Abcam; Cambridge, MA, USA), Texas red horse anti-mouse H+L IgG (1:500; Vector Laboratories; Burlingame, CA, USA), and Texas red goat anti-rabbit H+L IgG (1:500; Vector Laboratories; Burlingame, CA, USA). Finally, the sections were washed with PBS and mounted on glass slides and coverslips using VECTASHIELD (Vector Laboratories; Burlingame, CA, USA). The slices were analyzed under a multispectral confocal laser scanning microscope (TCS SPE; Leica; Heidelberg, GER) at excitation-emission wavelengths of 405–510 nm (Hoechst 33,258), 488–522 nm (Alexa 488) and 596–620 nm (Texas red). LAS AF software at 20x, 40x, and 63x (Leica Microsystems; Nussloch, GER) was used to acquire the images of the substantia nigra in four different anatomic levels (one rostral, two medials, and one caudal) per rat (*n* = 3 independent rats per group and time).

#### Immunohistochemistry

Immunohistochemistry was performed to accurately count dopaminergic neurons, activated microglia, and reactive astrocytes. First, endogenous peroxidase was depleted by a 3% hydrogen peroxide solution containing 0.3% Triton X-100 in PBS and 10% methanol at RT. The slices were incubated overnight with primary antibodies at 4 °C (Supplementary file 1: Table S2). The primary antibodies were a monoclonal anti-TH mouse (1:1000; Sigma-Aldrich; St. Louis, MO, USA), goat polyclonal anti-Iba1 (1:1000; Abcam; Cambridge, UK), a mouse monoclonal anti-GFAP Clone GA5 (1:500; Cell Signaling Technology; Danvers, MA, USA). Then, the slices were incubated for 2 h at RT with one of the following secondary antibodies: biotinylated horse anti-mouse IgG (1:300; Vector Laboratories; Burlingame, CA, USA) and biotinylated horse anti-goat IgG (1:300; Vector Laboratories; Burlingame, CA, USA). After washing with PBS, the slices were incubated for 2 h at RT with the avidin–biotin-peroxidase complex (ABC) kit (1:10; Vector Laboratories; Burlingame, CA, USA). The color was developed by adding diaminobenzidine solution containing H2O2 (DAB; Sigma-Aldrich; St. Louis, MO, USA). The sections were mounted on glass slides using Entellan (Merck KGaA; Darmstadt; GER) and examined with a Leica DMIRE2 microscope (Leica Microsystems; Nussloch, GER) in a bright field equipped using the 5x, 20x, and 40 × objectives. The images were digitized with a Leica DC300F camera (Leica Microsystems; Nussloch, GER). Negative controls were obtained by omitting the primary antibody or using the contralateral substantia nigra without transfection.

#### Densitometry and neuron counting

ImageJ-win64 software 1.52p (The National Institutes of Health; Bethesda, MD, USA) was used to measure the total TH, Iba1, and GFAP staining density in assays in immunohistochemistry assays. All background intensity was eliminated from the immunohistochemically stained area to quantify only the mean intensity of TH(+) cells in the substantia nigra and TH(+) fibers in the striatum. Iba1 and GFAP density were measured only in the substantia nigra. The final measurement was the mean value per nucleus and rat (*n* = 3 rats for every experimental condition).

The immunofluorescence area density (IFAD) was measured in the fluorescence assays using ImageJ software (The National Institutes of Health; Bethesda, MD, USA) in six levels per nucleus and per rat (*n* = 6 independent rats per experimental condition). The final value was the mean value calculated from the average of the six-level quantification per nucleus and rat.

#### Beam test

This study evaluates the motor coordination and walking speed when the rat travels on a narrow beam (2 m long, 1 cm wide, 30° tilt). All rats were trained 1 week before the behavior test on a wide beam (2 m long, 2 cm wide, 30° tilt) to acquire a behavior baseline of 6 s to transverse the beam. The rats were video-recorded during the trial to count the time across the beam and slips [59, 60].

#### Vibrissa evoked forelimb placement test

The left and right vibrissae of the rats were separately brushed against the edge of a table to evoke the forelimb positioning response. The two forelimbs were independently tested for ten trials by holding the rat by the torso to allow the limbs to hang freely, so neither limb nor tail supported any weight. The typical motor response consisted of a rapid and precise movement of the forelimb of the side stimulated that terminates on the table. The behavior was scored as a percentage of successful placement for limb (ipsilateral or contralateral to FUS application) per rat. The score = "0" means the absence of a response. The baseline behavior corresponds to the untreated control rats [23]. 

#### Test of asymmetry of forelimb use in a cylinder

Control, sham, and transfected rats were evaluated in a transparent acrylic cylinder (30 cm in height, 20 cm in diameter) to videotape the 20 first contacts on the cylinder wall made with the left and right forelimbs and both (simultaneously) [61]. The asymmetry was calculated as the percentage of contacts with the ipsilateral forelimb+1/2 of simultaneous contacts, divided by the total number of contacts (ipsilateral+contralateral+simultaneous) and the quotient multiplied by 100. The baseline behavior corresponds to the untreated control rats [59, 60, 62]. 

#### Statistical analysis

All results were expressed as mean ± standard deviation from at least 3 independent experiments (*n* = 3). The difference among the groups was analyzed with repeated measures of one-way ANOVA followed by a Tukey post hoc test for immunofluorescence results and immunohistochemistry for Iba1 and GFAP. In addition, two-way ANOVA followed by the Tukey post hoc test was used to analyze differences among the groups in TH(+) cells, TH(+) axonal projections, and behavioral tests. Graph Pad Prism 9.0.0 software (GraphPad Software Inc; La Jolla, CA, USA) was used for statistical analysis. Accepted significance was at *p* < 0.05.

## Results and discussion

### Physical characteristics of NTS-polyplex NPs

**Fig. 1:**
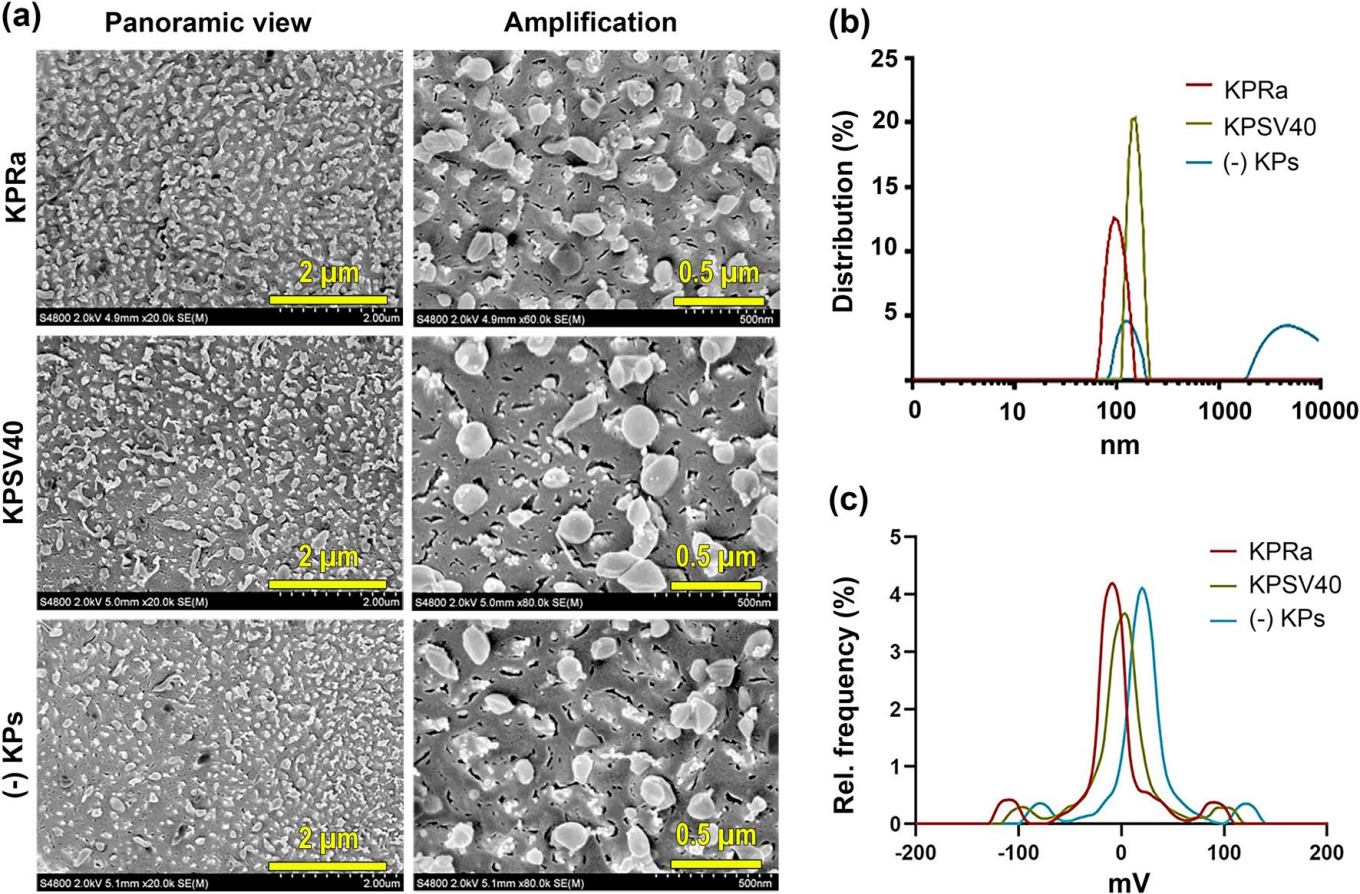
Size and Z-potential of NTS-polyplex nanoparticles harboring the plasmid pEGFP-N1. The nanoparticles were prepared with 6 nM pDNA, 144 nM NTS carrier, and 3 µM karyophilic peptides (KPRa or KPSV40) or without karyophilic peptides (-KPs). **a** Representative Cryo-electron microscopy (Cryo-SEM) micrographs of NTS-polyplex NPs. **b** Hydrodynamic radius of size distribution by Dynamic light scattering (DLS) and **c** Zeta potential by Electrophoretic light scattering (ELS)

Previous studies [4, 5, 11] suggest that features that block NTS-polyplex NPs from passing the BBB when injected via blood circulation are their size of around 100 nm, positive electrical charge, and the lack of specific transporters in the BBB [3–5, 11, 13]. Here, we explored the size and surface electrical charge of NTS-polyplex NPs resulting from compaction of the pEGFP-N1 plasmid with NTS carrier and with KPRa, KPSV40, or without KPs. Cryo-SEM studies showed spheroidal NPs with a mean diameter of 79.6 nm for KPRa, 102.7 nm for KPSV40, and 95.1 nm for NPs without KP (Fig. 1a and Table 1). DLS analysis showed average hydrodynamic radii of 65.0 nm for NPs with KPRa, 165.4 nm for KPSV40, and 130.9 nm without KP (Fig. 1b and Table 1). ELS showed mean Zeta-potential values of NTS-polyplex NPs of + 1.3 mV with KPRa, + 7.9 mV with KPSV40, and + 16.5 mV without KP, i.e., here a systematic increase of positive charging is observed in these complexes (Fig. 1c and Table 1). In studies on gene delivery via different routes, NPs with KPRa were tested due to their smaller size and lower positive electrostatic charge than those with KPSV40 after confirming the FUS-mediated aperture of BBB using EB as a tracer.

**Table 1:** Summary of dynamic light scattering analysis

NTS-polyplex NPs	Hydrodynamic radius (nm)	Polydispersity index (%)	Peak intensity 1	Mean zeta potential (mV)	Distribution peak value (mV)
With KPRa	65.0	52.8	93.5	+ 1.3	+ 3.4
With KPSV40	165.4	23.8	115.4	+ 7.9	+ 3.4
Without KPs	130.9	29.5	103.3	+ 16.5	+ 20.2

### FUS enables gene transfection of nigral dopaminergic neurons through blood and nasal pathways

**Fig. 2:**
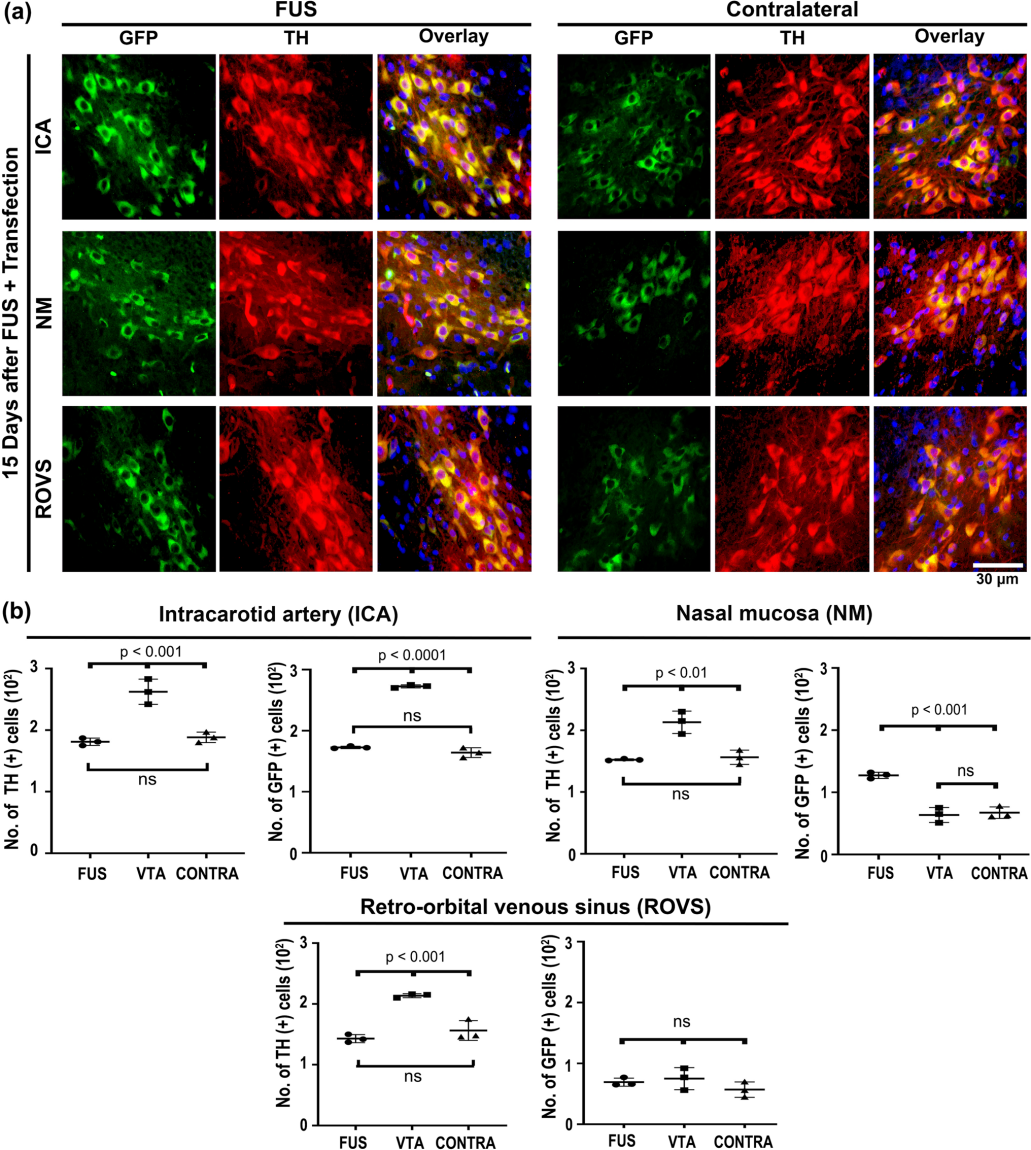
Focus ultrasound (FUS) enables the targeted green fluorescent protein (GFP) gene delivery to nigral dopamine neurons via different administration routes. Thirty minutes after FUS, 35 μL of NPs harboring the pEGFP-N1 plasmid were injected into the internal carotid artery, the retro-orbital venous sinus, or deposited through a capillary tube on the nasal mucosa. **a** Representative micrographs of the substantia nigra 15 days after transfection. The merged micrographs also include the Hoechst nuclear counterstaining. The value of the calibration bar is valid for all micrographs. **b** Quantification of GFP(+) cells and TH(+) cells using ImageJ software in the conditions shown by the micrographs of panel a). The values are the mean ± SD from three anatomical levels (*n* = 3 independent rats per experimental condition). GFP (green) immunoreactivity in TH (red) cells with nuclear Hoechst counterstaining (blue) in the substantia nigra. One-way ANOVA and post hoc Tukey tests. *ns* Not significant

**Fig. 3:**
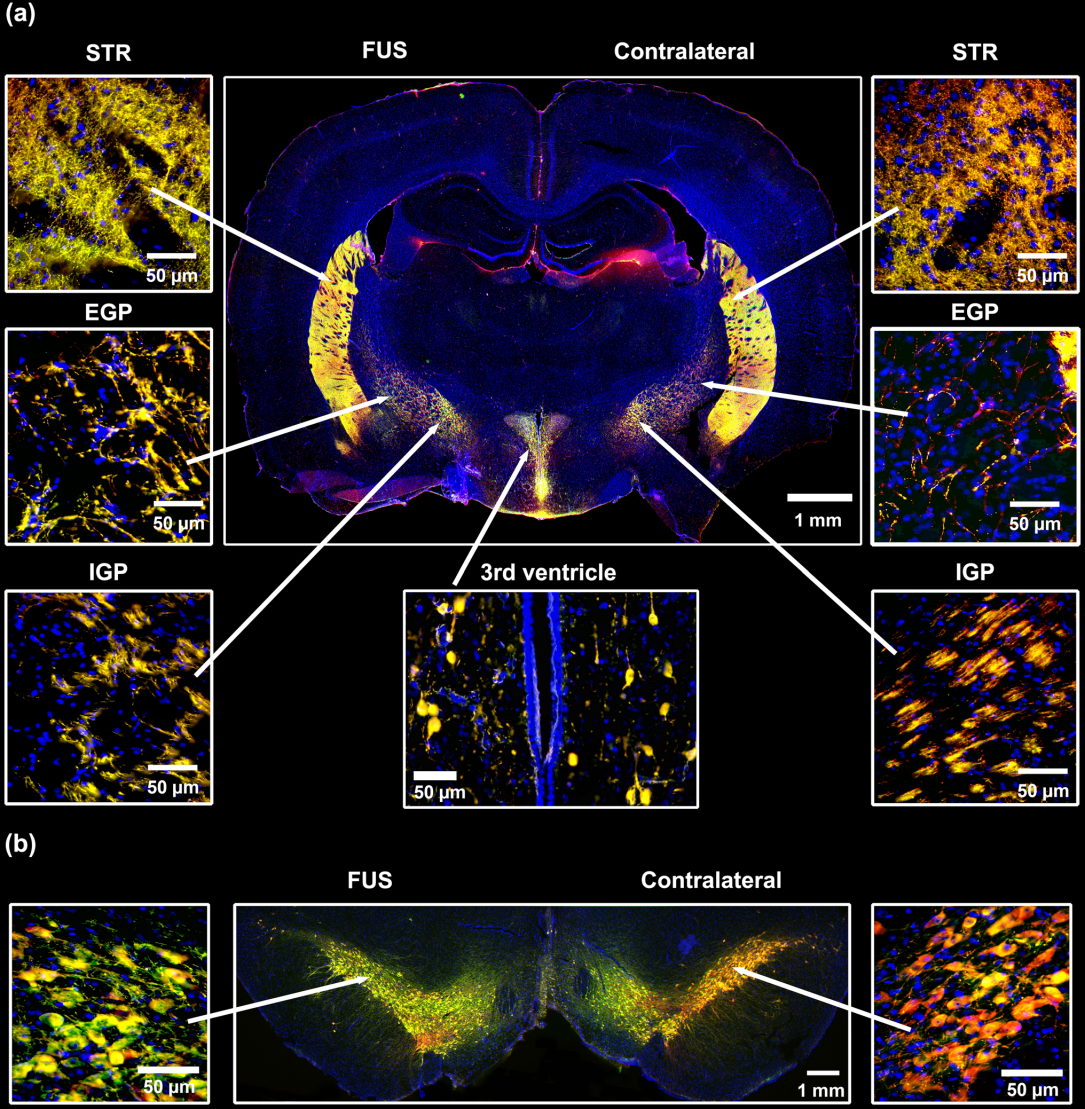
Bilateral distribution of green fluorescent protein (GFP) in dopaminergic cells and axonal ramifications in their target innervation nuclei after transfection via the internal carotid artery in FUS-treated rats. Representative micrographs 15 days after transfection of **a** coronal brain slice at − 2.16 mm from bregma and **b** mesencephalon slice immunostained against GFP and TH and counterstained with Hoechst. The amplifications show details of GFP expression in cells and axonal projections. *STR* Striatum, *EGP* External globus pallidus, *IGP* Internal globus pallidus. GFP (green) immunoreactivity in TH (red) cells with nuclear Hoechst counterstaining (blue). The scale value is equal for the respective set of micrographs

Experiments with EB administration through different blood routes revealed a blue area in the substantia nigra where FUS was applied, thus demonstrating EB extravasation because of BBB opening (Supplementary file 1: Fig. S3). This effect occurred regardless of the administration route; however, the internal carotid artery supplied the highest EB density in the FUS-treated substantia nigra with a less EB volume (75 μL) than the retro-orbital sinus (150 μL) and caudal vein (300 μL); the intravenous injection routes were similarly effective since they produced a comparable density of EB in the substantia nigra (Supplementary file 1: Fig. S3), as was previously demonstrated by another research group [63]. No EB extravasation occurred in the brain when the dye was administered via nasal mucosa after FUS, possibly due to the absence of a transport mechanism for EB in agreement with previous reports in healthy rats [64]. In contrast, no blue color was observed when EB was administered without FUS, as shown previously [44]. These experiments indicate that the shortest route to reach the substantia nigra would optimize the dose of NPs for gene delivery. This hypothesis was explored by administering an equal amount of NTS-polyplex NPs (1.69 µg pDNA/35 μL DMEM) via the internal carotid artery, retro-orbital venous sinus, or nasal mucosa. Fifteen days after transfections, double immunofluorescence assays showed GFP expression in dopaminergic neurons of both the FUS-treated substantia nigra and the untreated contralateral (CONTRA) substantia nigra with different efficiency depending on the route administration (Fig. 2). The delivery through an internal carotid artery or retro-orbital venous sinus caused similar GFP expression in TH(+) cells in the left and right substantia nigra (Fig. 2); however, GFP expression in TH(+) neurons in the contralateral substantia nigra was 63.13% less than in the ipsilateral side (Fig. 2). Transfection in the contralateral side cannot be explained by BBB opening on this side because FUS was applied in the opposite substantia nigra. The bilateral transfection after arriving NTS-polyplex NPs into the FUS-treated substantia nigra is likely due to the unique feature of diffusing to the contralateral side after their unilateral administration, as recently shown in healthy and parkinsonian rats [7]. When comparing the percentage of dopaminergic neurons expressing GFP in the ipsilateral and contralateral substantia nigra among the three delivery routes, it was found that the highest transfection occurred by the internal carotid artery delivery (FUS, 87.5%, ventral tegmental area (VTA), 97.2%, CONTRA, 73.3%), followed by the intranasal route (FUS, 61.3%, VTA, 30.5%, CONTRA, 24.4%), and then by the retro-orbital sinus (FUS, 47.1%, VTA, 28.0%, CONTRA, 13.6%) (Fig. 2). These results agree with the shorter arterial irrigation of the substantia nigra. Thus, the internal carotid artery irrigates it immediately through the basilar and posterior cerebral arterial branches [65]. On the contrary, the retro-orbital venous sinus blood reaches the substantia nigra as arterial blood after passing by the superior cava vein, heart, and lungs to return to the heart and then continue by the internal carotid artery and branches [65]. After passing through these organs, some NTS-polyplex NPs are trapped, thus decreasing the effective concentration [13]. Of the three paths proposed for nasal delivery to the brain [66], the retrograde transport of NTS-polyplex NPs by the olfactory and trigeminal nerves can be ruled out because no GFP expression was observed in control rats without FUS treatment (Supplementary file 1: Fig. S4). Their delivery can likely be by the circulation of the submaxillary lymphatic system or nasal veins, which go into the superior cava vein through respective collecting vessels to finally arrive into the substantia nigra by collaterals of internal carotid artery similar to retro-ophthalmic venous sinus [66]. The internal carotid artery route also led to high production of GFP, which spread to the innervation nuclei, including the striatum, internal and external globus pallidus, and subventricular zone of the third ventricle of both cerebral hemispheres (Fig. 3 and in Supplementary file 1: Fig. S5). The proper controls of immunofluorescence for GFP and TH ensure the validity and reliability of the FUS results (Supplementary file 1: Fig. S6). Therefore, the presence of GFP in dopaminergic neurons and axonal projections proves that FUS overcame BBB blockage and preserved NTS-polyplex NPs specificity for dopaminergic neurons [4, 6, 17, 22, 23]. Because the plasmid pEGFP-N1 lacks a cell-specific promoter, the specificity of transfection is provided by the NTS ligand, which internalizes NTS-polyplex NPs through endocytosis of NTSR1, highly expressed in dopaminergic neurons, as previously demonstrated in primary neuron cultures and animals [2, 8, 9, 15, 16]. The absence of GFP fluorescence in the substantia nigra in animals without FUS application but injected with NTS-polyplex NPS via the intracarotid artery discards the possibility of autofluorescence (Supplementary file 1: Fig. S4). Besides, our results support and expand the finding that FUS can also facilitate the targeted delivery of genetic material to dopaminergic neurons, even when the gene vectors lack specificity for these neurons [32, 37–39, 67].

The administration via the internal carotid artery led to the most efficient gene expression, followed by administrations through the nasal mucosa and then the retro-orbital vein at the same dose injected. In the first transfection modality, the high gene expression in nigral dopamine neurons allowed GFP to be distributed by the axonal terminals to the target innervation nuclei of dopaminergic neurons. Moreover, the transfection occurred in nigral dopaminergic neurons of both brain sides according to the bilateral gene expression caused by a unilateral intracerebral transfection [7]. This feature of NTS-polyplex NPs represents a competitive advantage for gene therapy compared to AAV gene delivery, which only causes gene expression on the FUS-treated side [28]. Similarly, EB only permeates on the side treated by FUS [44]. Ongoing studies are devoted to exploring whether microsome-mediated transport or glymphatic circulation is the mechanism of NTS-polyplex NP diffusion.

### Transient activation of neuroinflammation

**Fig. 4:**
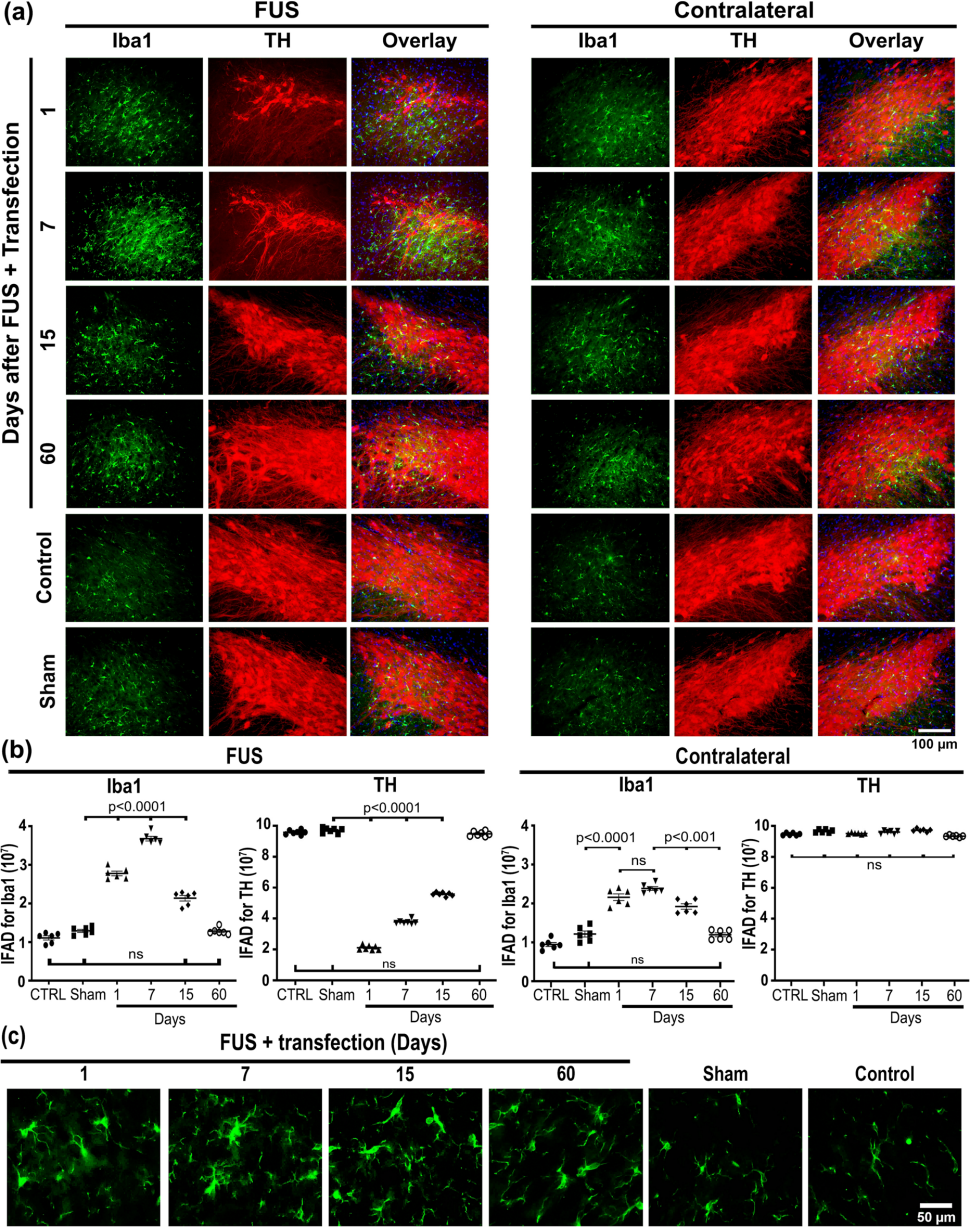
Focus ultrasound (FUS) causes transient microglia activation in the substantia nigra. **a** Representative micrographs of the most injured site (where FUS was applied) of the substantia nigra double immunostained with positive to ionized calcium-binding adaptor molecule 1 (Iba1) and tyrosine hydroxylase (TH) over time after FUS. The merged micrographs also include the Hoechst nuclear counterstaining. **b** Area density (IFAD) for Iba1 and TH immunofluorescence measured from panel **a** micrographs using ImageJ software. The values are the mean ± SD from three anatomical levels (*n* = 6 independent rats per experimental condition). One-way ANOVA and post hoc Tukey tests. *ns* Not significant. Iba1 (green) immunoreactivity in TH (red) cells with nuclear Hoechst counterstaining (blue) in the substantia nigra over time. **c** Amplified micrographs show Iba1(+) cells with phenotypic changes according to their activation state. The scale value is equal for the respective set of micrographs

Although neuroinflammation is the primary expected response to BBB-forced disruption by FUS [51], it remains less explored, especially the long-term noxious impact on the substantia nigra, a nucleus enriched with microglia cells in physiological conditions [50]. Previous works have shown that FUS may result in temporary neuroinflammation in the cerebral cortex, comparable to ischemia or mild traumatic brain injury [68]. Therefore, we utilized the untreated contralateral substantia nigra, which also shows GFP expression, to examine any possible damage by transfection. In agreement with the harmful effect [68], FUS transiently increased Iba1(+) basal values to a maximum of 184.8% on day 7 that declined later and normalized on day 60 after FUS in the treated substantia nigra compared with the control and sham groups, thus showing reversible microglia activation (Fig. 4 and in Supplementary file 1: Fig. S7). On the contrary, IFAD for TH(+) cells decreased by 80% 24 h after FUS compared with the control and sham groups, but it reached basal values on day 60 (Fig. 4). In the contralateral substantia nigra, there was also a rise in Iba1(+) basal values but 50% less than on the FUS-treated side and also reversible after day 7 post-FUS. However, TH(+) cells were unaffected, thus suggesting no harmful effect of transfection (Fig. 4).

Immunohistochemistry analysis of Iba1 showed a similar activation profile to immunofluorescence results but with higher counts because the quantification included the complete substantia nigra (Supplementary file 1: Fig. S7). Amplifications showed no evidence of advanced stages of microglial activation, such as phagocytic forms, at the activation peak (Fig. 4c and in Supplementary file 1: Fig. S7). Therefore, severe parenchymal damage due to neuroinflammation can be ruled out because it occurs when activated microglia acquire a phagocytic state [69–72]. Nevertheless, the induction of neurotoxic A1 astrocytes from the reactive astrocyte population was explored because this phenomenon follows microglial activation [42]. FUS also caused reactive astrogliosis in the substantia nigra exhibited by increased GFAP immunoreactivity (Supplementary file 1: Fig. S8).

Since GFAP immunoreactivity in healthy conditions is imperceptible in the substantia nigra pars compacta [69–71], GFAP(+) cells were first localized there using TH immunofluorescence staining, which reveals dopaminergic neurons, and then the astrocyte subtypes were identified over time after FUS (Supplementary file 1: Fig. S8).

**Fig. 5:**
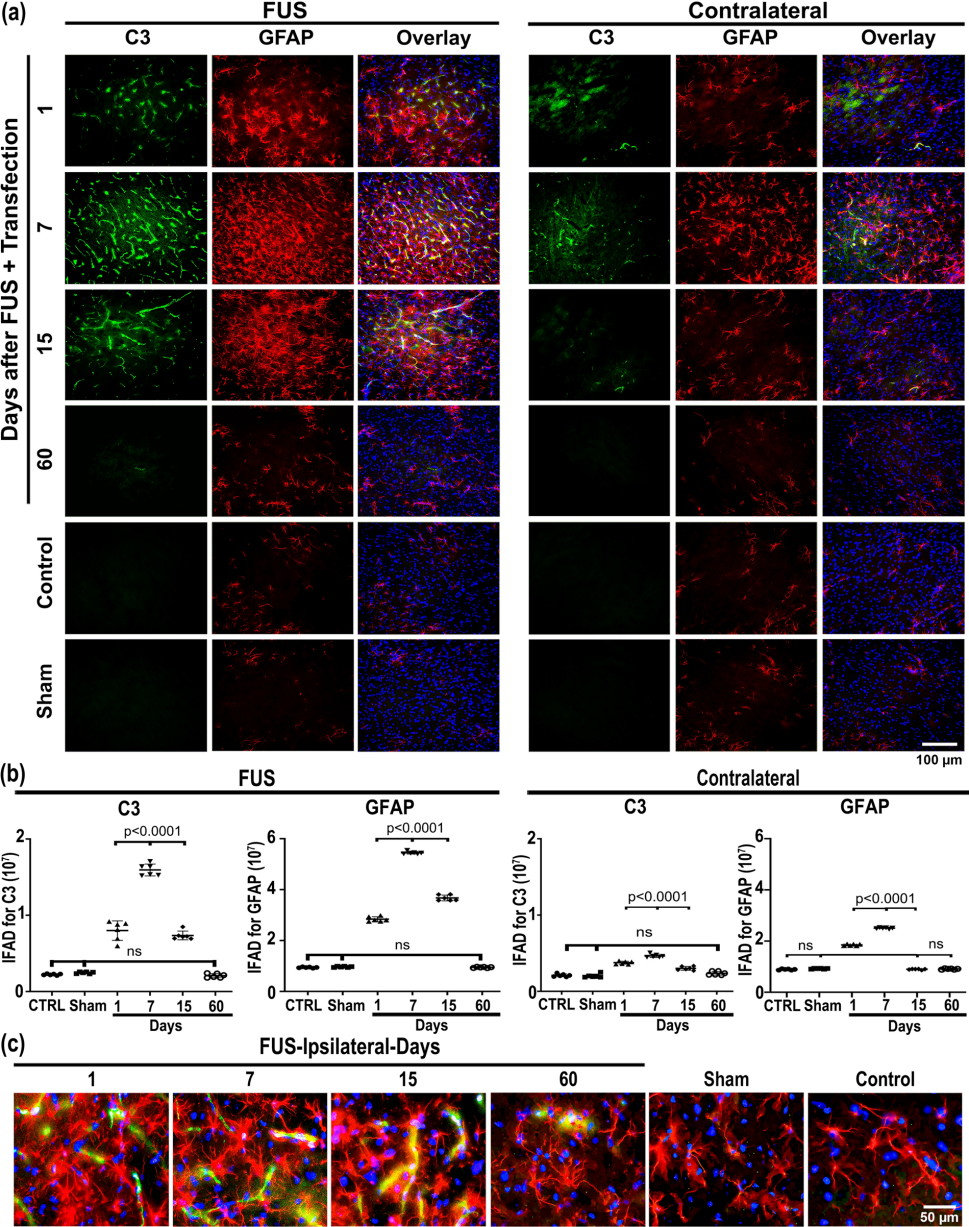
Transient induction of neurotoxic A1 astrocyte after FUS and gene delivery. **a** Representative micrographs of the most injured site of the substantia nigra double immunostained with complement 3 (C3) and glial fibrillary acidic protein (GFAP) over time after FUS. The merged micrographs also include the Hoechst nuclear counterstaining. **b** C3 and GFAP immunofluorescence area density (IFAD) measured from micrographs of panel A using ImageJ software. The values are the mean ± SD from three anatomical levels (*n* = 6 independent rats per experimental condition). One-way ANOVA and post hoc Tukey tests. *ns* Not significant. **c** Amplified merged images show the reduction of C3 (green) immunoreactivity in GFAP(+) (red) cells with nuclear Hoechst counterstaining (blue) in the substantia nigra over time. The scale value is equal for the respective set of micrographs

**Fig. 6:**
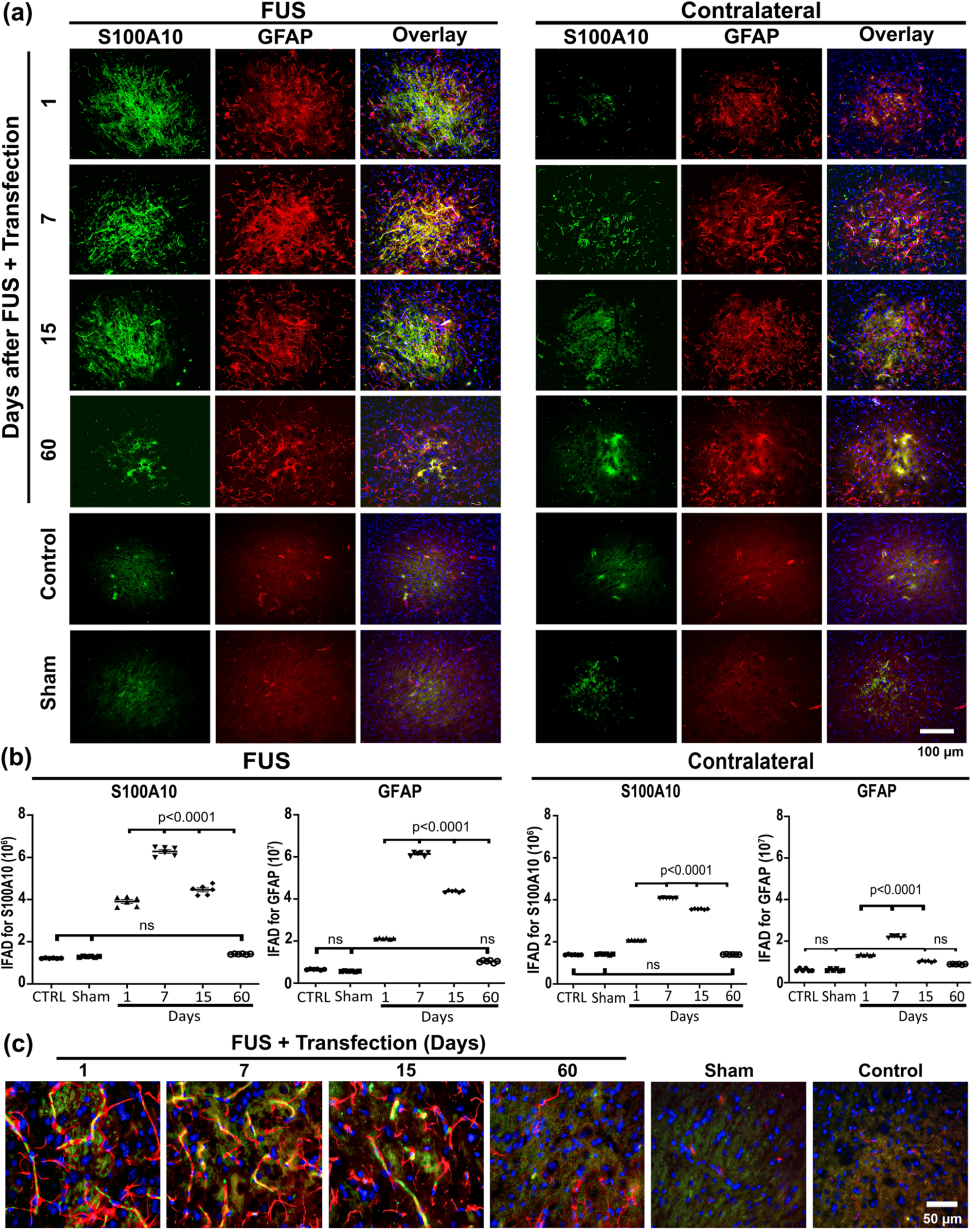
Transient neurotrophic A2 activation after focus ultrasound (FUS) and gene delivery. **a** Representative micrographs of the most injured site of the substantia nigra double immunostained with calcium-binding protein A10 (S100A10) and GFAP over time after FUS. The merged micrographs also include the Hoechst nuclear counterstaining. **b** S100A10 and GFAP immunofluorescence area density (IFAD) measured from micrographs of panel A using ImageJ software. The values are the mean ± SD from three anatomical levels (*n* = 6 independent rats per experimental condition). One-way ANOVA and post hoc Tukey tests. *ns* Not significant. **c** Amplified merged images show the reduction of S100A10 (green) immunoreactivity in GFAP (red) cells with nuclear Hoechst counterstaining (blue) in the substantia nigra over time. The scale value is equal for the respective micrograph set

FUS increased the double GFAP-C3(+) cell population, which followed a similar time course to activated microglia in the treated substantia nigra and on the contralateral untreated side, thus showing that induction of neurotoxic A1 astrocytes was also reversible (Fig. 5). However, the magnitude of neurotoxic A1 astrocyte activation was 173.1% higher on the ipsilateral side than on the contralateral substantia nigra, suggesting that transfection had a negligible effect on inducing neurotoxic A1 astrocytes (Fig. 5). A significant transient increase in double S100A10-GFAP(+) cells was also induced in the treated and contralateral substantia nigra after FUS (Fig. 6) that followed the time course of double C3-GFAP(+) cells (Fig. 5), thus showing that neurotrophic astrocytes A2 were also induced. Again, the increase in S100A10-GFAP(+) cells was significantly 453.2% higher in the FUS-treated substantia nigra than on the contralateral untreated side (Fig. 6). The finding that neurotoxic A1 astrocyte activation was reversible and the contrary increase in neurotrophic A2 astrocytes suggests that neuronal damage was minor and could be reparable by the action of neurotrophic factors released by the neurotrophic astrocytes [6, 73, 74].

Immunohistochemistry analysis of GFAP(+) cells displayed the reactive astrocytosis elicited by FUS that followed the time course of neurotoxic astrocytes A1 and neurotrophic astrocytes A2 revealed by immunofluorescence (Figs. 5 and 6 and in Supplementary file 1: (Fig. S8 and S9)). Therefore, those astrocyte populations could come from the reactive astrocytes, as shown previously in purified astrocyte cultures [42].

**Fig. 7:**
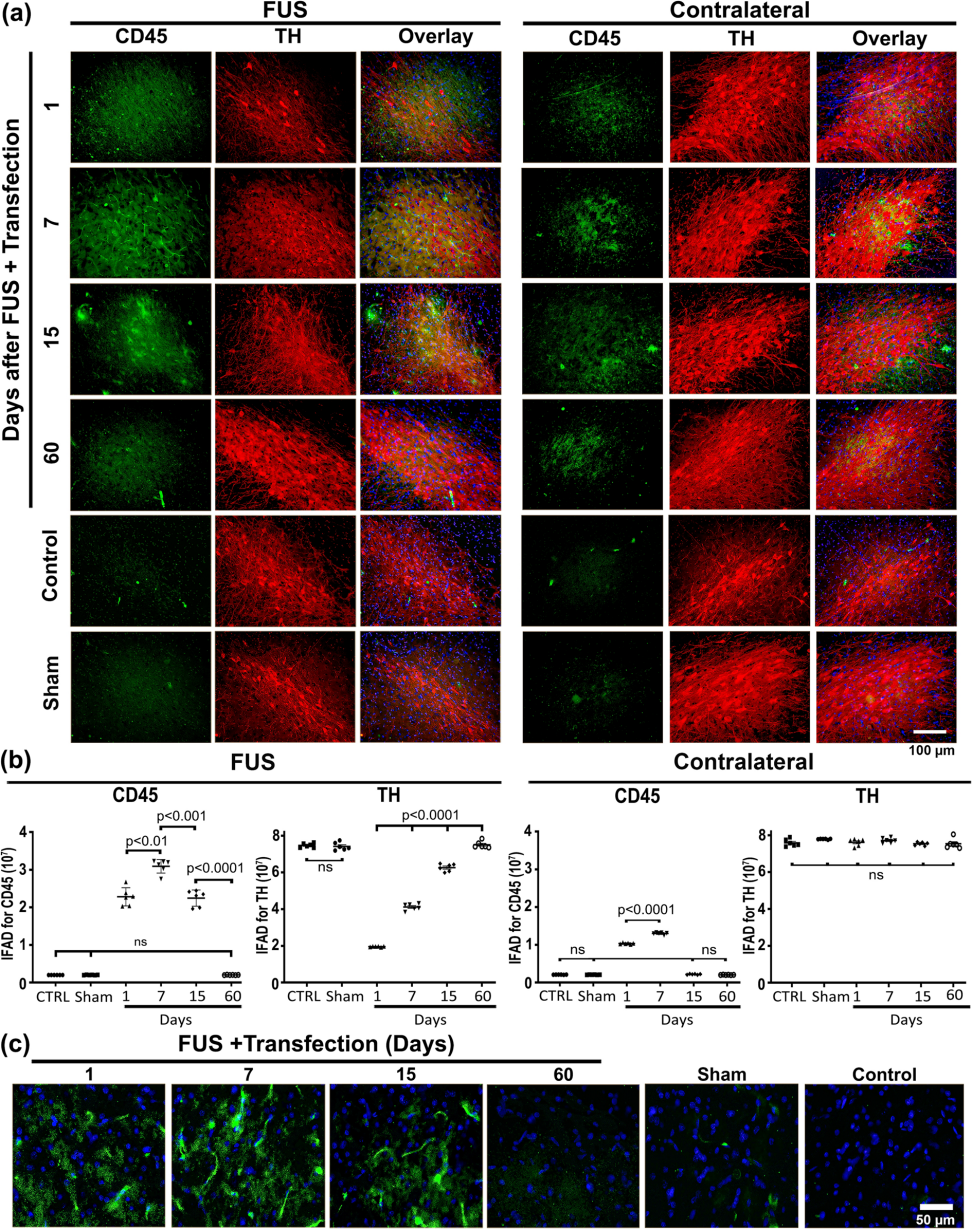
Transient leucocyte infiltration after focus ultrasound (FUS) and gene delivery. **a** Representative micrographs of the substantia nigra with double immunostaining against the cluster of differentiation CD45 and tyrosine hydroxylase (TH) over time after FUS. The merged micrographs also include the Hoechst nuclear counterstaining. **b** CD45 and TH immunofluorescence area density (IFAD) measured from micrographs of panel A using ImageJ software. The values are the mean ± SD from three anatomical levels (*n* = 6 independent rats per experimental condition). One-way ANOVA and post hoc Tukey tests. *ns* Not significant. CD45 (green) immunoreactivity in TH (red) cells with nuclear Hoechst counterstaining (blue) in the substantia nigra over time. **c** Amplified micrographs show morphological details of CD45(+) cells with nuclear counterstaining and their disappearance after 60 days post-FUS. The scale value is equal for the respective micrograph set

The presence of CD45(+) cells in the FUS-treated substantia nigra demonstrated lymphocyte infiltration (Fig. 7), thus reinforcing the finding of BBB aperture shown by EB administration (Supplementary file 1: Fig. S3). Compared with the control values, the increase in CD45(+) cells was 1022.1% on the first day and reached the maximum increase of 1421.2% on day 7 after FUS. Later, CD45(+) cells decreased to 105.1% on day 15 and came to basal values at the end of the study (60 days). CD45(+) cells also increased in the contralateral untreated substantia nigra, but in less percentage than on the FUS-treated side, reaching a maximum increment of 880.4% on day 7. TH(+) cells also decreased until day 7, then recuperated, and remained unaffected on the contralateral untreated side (Fig. 7), in agreement with the results shown in Fig. 4. Since CD45 is long known to be preferentially expressed in T lymphocyte activation, its disappearance after day 60 post-FUS rules out the evolution to chronic neuroinflammation by the professional immune response participation, which contradicts the proposal of previous work [75]. A difference in the experimental approach can explain the discrepancy because the latter work used FUS combined with microbubbles, whereas we used only FUS.

### Effect of FUS and transfection on the nigrostriatal system and motor behavior

**Fig. 8:**
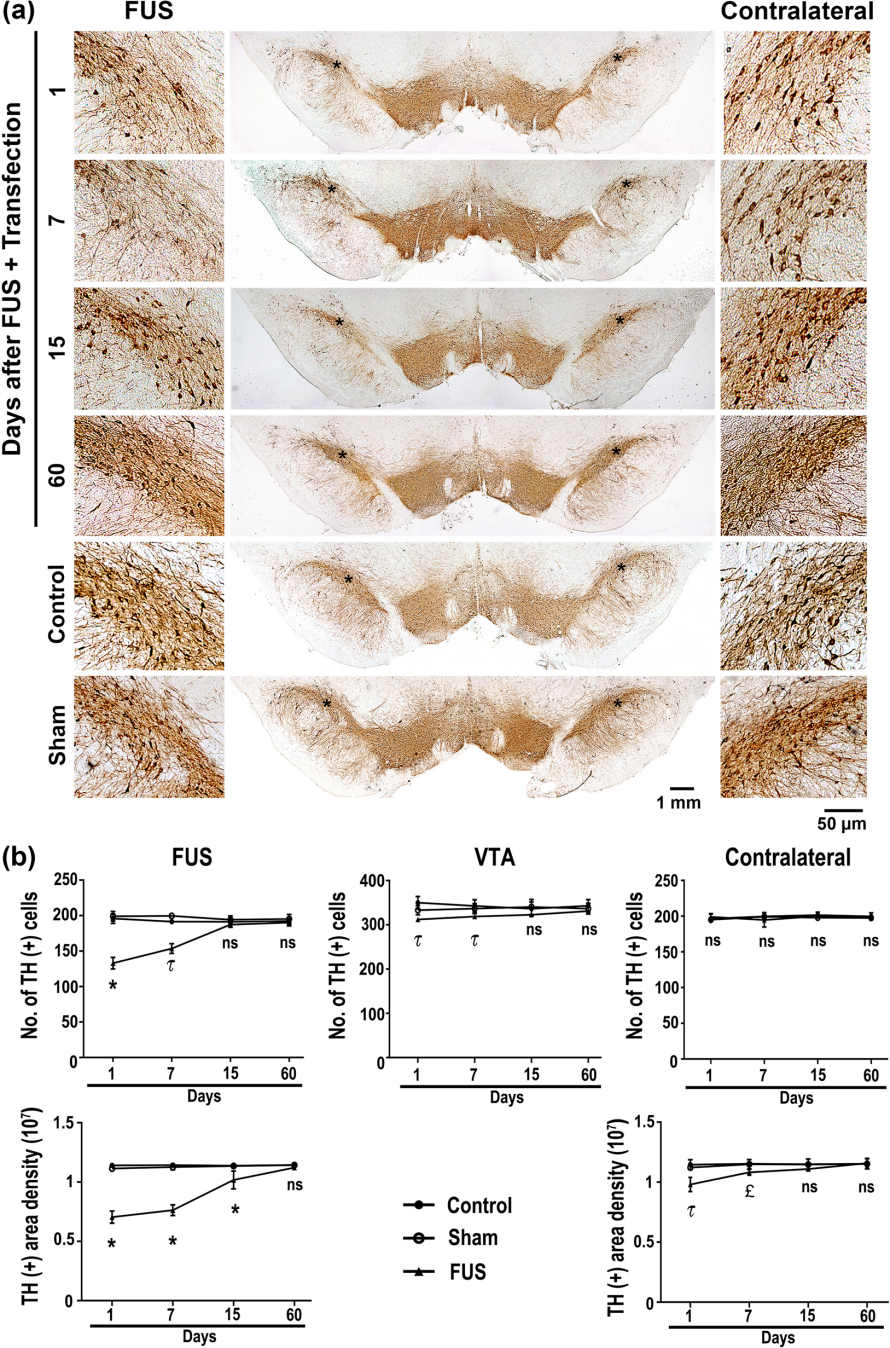
Reversible drop of dopaminergic neuron population of the substantia nigra after focus ultrasound (FUS) and transfection. **a** Representative micrographs of the substantia nigra with tyrosine hydroxylase (TH) immunohistochemistry over time after FUS. The scale value is equal for the respective set of micrographs. **b** TH(+) cell counting and **c** immunoreactivity area density measured from micrographs of panel **a** using ImageJ software. The values are the mean ± SD from average measurements in five anatomical levels (*n* = 3 independent rats per experimental condition. *p* < 0.0001 = *, *p* < 0.001 = τ and *p* < 0.01 = £ compared with the control and sham groups in each time-point. Two-way ANOVA and post hoc Tukey tests. *ns* Not significant

**Fig. 9:**
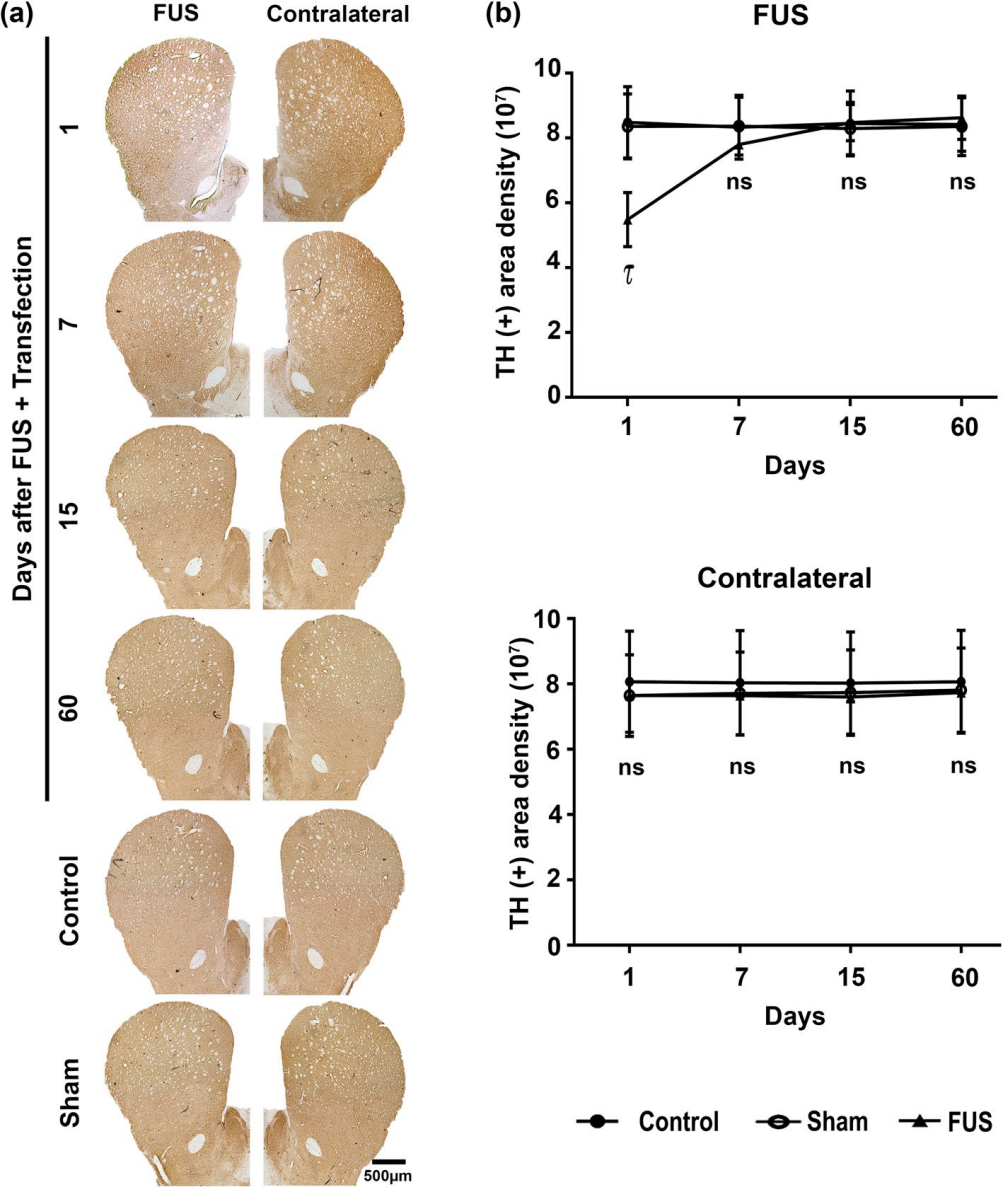
Reversible decrease in dopaminergic innervation of the striatum after focus ultrasound (FUS) and transfection. **a** Representative micrographs of the striatum with tyrosine hydroxylase (TH) immunohistochemistry over time after FUS. The scale value is equal for all micrographs. **b** TH(+) immunoreactivity density from micrographs of panel **a** using ImageJ software. The values are the mean ± SD from the average measurements in five anatomical levels per rat (*n* = 3 independent rats per experimental condition). *p* < 0.001 = τ, compared with the control and sham groups at each time point. Two-way ANOVA and post hoc Tukey tests. *ns* Not significant

Although FUS is considered a non-/minimally invasive approach, collateral undesired effects, such as cerebral hemorrhage [76, 77], have been reported even with low intensity mainly because of the intracerebral thermal rise during the wave traveling and significantly in the focus site [78, 79]. Therefore, immunohistochemistry assays have been performed to quantify the FUS impact on nigral dopaminergic neurons and striatal axonal projections more accurately and confirm the immunofluorescence results (Figs. 4 and 6). On the first day, the FUS-treated substantia nigra had a 37.5% depletion of TH(+) cells and their branching (Fig. 8). Then, a significant recovery in TH(+) cells and branching followed until it was complete on day 15 (Fig. 8). However, a significant 40% drop in TH(+) density in the striatum occurred only on the first day after FUS (Fig. 9). There were no evident harmful effects on the dopaminergic nigrostriatal pathway of the contralateral untreated side, as shown by the lack of significant changes in TH(+) cells and branching of the substantia nigra (Fig. 8) and TH(+) density in the striatum (Fig. 9).

The nigral dopaminergic neurons are particularly vulnerable to neuroinflammation due to their poor glutathione levels, reduced glutamylcysteine ligase activity, natural antioxidant defenses in neuronal cells [80], and the enriched microglial population [50]. The poor antioxidant defense explains the weak depuration of the nonenzymatically oxidized products of dopamine and the oxidants resulting from the iron-dependent Fenton reaction [81]. The enriched microglia population favors a fast response to the minimum oxidative stress imbalance [69–71], which can account for the immediate neuroinflammation response to the transiently increased temperature of 4 °C for 2.5 min produced in our FUS condition [44]. Therefore, it is plausible that the reversible decrease in the dopaminergic neuron population could come from a temporary loss of metabolic function caused by the heat-induced homeostasis disbalance rather than the harmful effects of neuroinflammation through neurotoxic A1 astrocytes and proinflammatory mediators. Furthermore, neurotrophic factors such as BDNF [73], GDNF [74], and CDNF [6], known to be released from astrocytes, can also contribute to the recovery of the dopaminergic nigrostriatal system.

Several reports using diverse sonication parameters with or without microbubbles agree that acute neuroinflammation displayed by activated microglia, reactive astrocytes, infiltrated leukocytes, proinflammatory cytokines, and chemokines occurs in different brain areas, particularly in the cerebral cortex and hippocampus. Intracellular signaling pathways, including AKT and NF-κB signaling, have also been demonstrated in acute neuroinflammation after FUS [78, 82, 83]. However, the long-term effect of neuroinflammation on neuronal survival remained poorly characterized. Our results in the substantia nigra showed reversible microglia activation without presenting phagocytic ameboid forms, thus indicating the absence of significant neurological damage despite the early reversible reduction of dopaminergic neurons in the FUS application site. This suggestion is supported by the finding that no neuronal affection is seen without phagocytic microglia form [69–71]. In addition, the increased activated microglia in the contralateral substantia nigra did not also produce significant neuronal damage. Consistently with activated microglia, neurotoxic A1 reactive astrocytes also occurred significantly in the FUS-treated substantia nigra and less in the contralateral side, suggesting that A1 astrocytes were induced by activated microglia through the well-known mechanism that involves proinflammatory cytokines and C1q [42, 70]. It could be thought that neurotoxic A1 astrocytes are responsible for the drop of dopaminergic neurons due to their known ability to kill neurons [42, 70, 71]. However, the maximum increase in neurotoxic A1 astrocytes occurred when dopaminergic neurons were recuperating. In contrast, neurotrophic A2 astrocytes, which also progressively rose from the first day after FUS, could help overcome the noxious effect of neurotoxic A1 astrocytes by releasing neurotrophic factors such as BDNF [73]. This suggestion is supported by findings that FUS stimulation in healthy mice can increase BDNF levels, which can induce neuronal recovery.

**Fig. 10:**
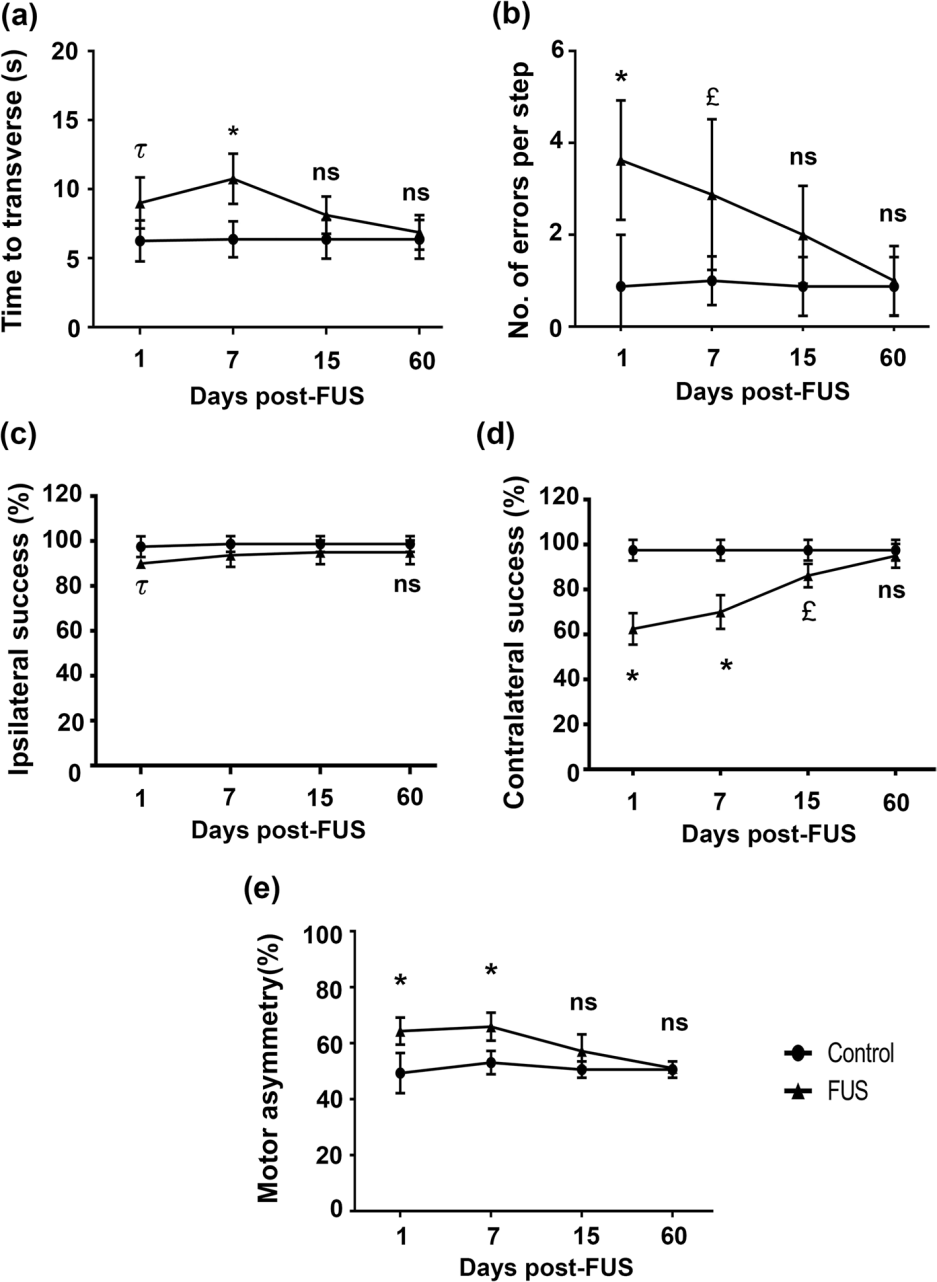
Reversible sensorimotor deficits after FUS and transfection. Rats were subjected to focus ultrasound (FUS) and transfected with NTS-polyplex NPs with pEGFP-N1 at 30 min after FUS and evaluated over time with the behavioral tests. **a** and **b** Beam walking test, **c** and **d** Vibrissae-evoked forelimb placing test, **e** Cylinder test. Values are expressed as the mean ± SD of 6 independent rats for each experimental condition. *p* < 0.001 = £, *p* < 0.01 = τ and *p* < 0.05 = *. Control group compared with FUS group. Two-way ANOVA and Tukey post hoc test. *ns* Not significant

The analysis of the four behavioral tests showed that the rats who underwent FUS treatment showed locomotor deficits, specifically during the decrease phase of the dopaminergic nigrostriatal system, compared with the control group (Fig. 10). The FUS-treated rats traveled the beam slower than the control group, with delays of 27.5% on day 1 and 68.6% on day seven and increased foot slips on days 1 (314.3%) and 7 (187.5%) (Fig. 10a and b). Compared with the control group, the vibrissae test showed a reduction in the forelimb place response of 10% on the ipsilateral side and 35.9% on the contralateral side to the FUS application that reached basal values on day 60 post-treatment (Fig. 10c and d). Finally, the cylinder test showed an asymmetry on day 1 (30.4%) and day 7 (24.1%) in using forelimbs to touch the cylinder wall, compared with the control groups (Fig. 10e). All rats showed normal behavior from day 15 post-FUS, indicating that the behavioral deficits were spontaneously reversible and correlated with the recovery of the dopaminergic nigrostriatal system (Figs. 8 and 9). Therefore, the reversible decrease in motor behavior could be associated with the decline of dopamine metabolism and an ineffective neuronal response rather than neuronal death, as found in another study [84]. This proposal is further supported by the finding that motor alterations were reversible and minor than those caused by neurotoxin-induced dopaminergic neuron death [4, 71].

## Conclusion

This study shows that gene delivery by NTS-polyplex NPs to the substantia nigra via blood and nasal pathways is feasible using FUS conditions that provide a 6-h window opening of BBB [44], thus eliminating the need for intracerebral administration. The gene expression in nigral dopaminergic neurons occurs, although NTS-polyplex NPs are supplied at the onset of acute neuroinflammation (60 min after FUS). Of the three pathways explored, the intracarotid administration is the most effective route. Neuroinflammation is reversible, but during its critical phase, decreased dopaminergic cells and locomotor behavior deficits occurred but returned to normal parameters 15 days later, suggesting a temporary loss of dopaminergic phenotype rather than neuronal death. These results demonstrate the safety of FUS in systemic transfections of an innocuous reporter gene. Nevertheless, confirming the safety of therapeutic gene expression in Parkinsonian animal models is necessary to advance this procedure to the clinic, especially in neurodegenerative disorders related to dopamine dysfunction.

### Supplementary Information

**Additional file 1 **Experimental design, FUS setup, Antibodies for double immunofluorescence staining, Antibodies for immunohistochemistry staining, Evans Blue delivery to show FUS-induced BBB opening, NTS-polyplex NPS via the intracarotid artery and nasal mucosa without FUS application, Identification of nuclei innervated with GFP(+) axonal terminals, Controls of double immunofluorescence, Reversible microglia activation, Reversible reactive astrogliosis and Localization of transient astrogliosis in the substantia nigra.

## Data Availability

The authors declare that the data supporting the findings of this study are available within the paper and its additional files. Should any data files be needed in any other format, they are available from the corresponding author upon reasonable request.
