# Supplementary material for: Focused ultrasound on the substantia nigra enables safe neurotensin-polyplex nanoparticle-mediated gene delivery to dopaminergic neurons intranasally and by blood circulation
Source: Discov Nano. 2024 Apr 2;19(1):60. doi: 10.1186/s11671-024-04005-9 (PMC10987469; doi:10.1186/s11671-024-04005-9)
Supplement: Supplementary file 1 — Additional file 1 Experimental design, FUS setup, Antibodies for double immunofluorescence staining, Antibodies for immunohistochemistry staining, Evans Blue delivery to show FUS-induced BBB opening, NTS-polyplex NPS via the intracarotid artery and nasal mucosa without FUS application, Identification of nuclei innervated with GFP(+) axonal terminals, Controls of double immunofluorescence, Reversible microglia activation, Reversible reactive astrogliosis and Localization of transient astrogliosis in the substantia nigra. [file 11671_2024_4005_MOESM1_ESM.docx]

**Focused ultrasound on the substantia nigra enables safe neurotensin-polyplex nanoparticle-mediated gene delivery to dopaminergic neurons through intranasally and blood circulation.**

Juan U. Mascotte-Cruz^1^, Arturo Vera^2, ‡^, Lorenzo Leija^2, ‡^, Francisco E. Lopez-Salas^3^, Michael Gradzielski^4^, Joachim Koetz^5^, Bismark Gatica-García^1,6^, CP Rodríguez-Oviedo^6^, Irais E. Valenzuela-Arzeta^1^, Lourdes Escobedo^1^, David Reyes-Corona^6^, ME. Gutierrez-Castillo^7^, Minerva Maldonado-Berny^1^, Armando J. Espadas-Alvarez^7^, Carlos E. Orozco-Barrios^8^, Daniel Martinez-Fong^1,6, ‡,*^

*^1^ Centro de Investigación y de Estudios Avanzados, Departamento de Fisiología, Biofísica y Neurociencias, Ciudad de México, México.*

*^2^ Centro de Investigación y de Estudios Avanzados, Departamento de Ingeniería Eléctrica-Bioelectrónica, Ciudad de México, México.*

*^3^ , Universidad Nacional Autónoma de México, Instituto de Investigaciones Biomédicas, Departamento de Biología Molecular y Biotecnología Ciudad de México, México.*

*^4^ Technische Universität Berlin, Institut für Chemie, Stranski-Laboratorium für Physikalische und Theoretische Chemie, Berlin, Germany.*

*^5^ Universität Potsdam, Institut für Chemie, Potsdam, Germany.*

*^6^ Nanoparticle Therapy Institute, Aguascalientes, México.*

*^7^ Instituto Politécnico Nacional, Centro Interdisciplinario de Investigaciones y Estudios sobre Medio Ambiente y Desarrollo, Departamento de Biociencias e Ingeniería, Ciudad de México, México.*

*^8^ CONAHCYT - Unidad de Investigaciones Médicas en Enfermedades Neurológicas, Hospital de Especialidades “Dr. Bernardo Sepúlveda. Centro Médico Nacional Siglo XXI, Instituto Mexicano del Seguro Social, Ciudad de México, México.*

*^‡^ These authors participated equally.*

** Correspondence should be addressed to Daniel Martinez-Fong, Departamento de Fisiología, Biofísica y Neurociencias, Centro de Investigación y de Estudios Avanzados, Av. Instituto Politécnico Nacional, No. 2508, San Pedro Zacatenco 07360 Ciudad de México, México. Telephone: +52+5557473959;* [*daniel.martinezfong@cinvestav.mx*](mailto:daniel.martinezfong@cinvestav.mx)*.*

**
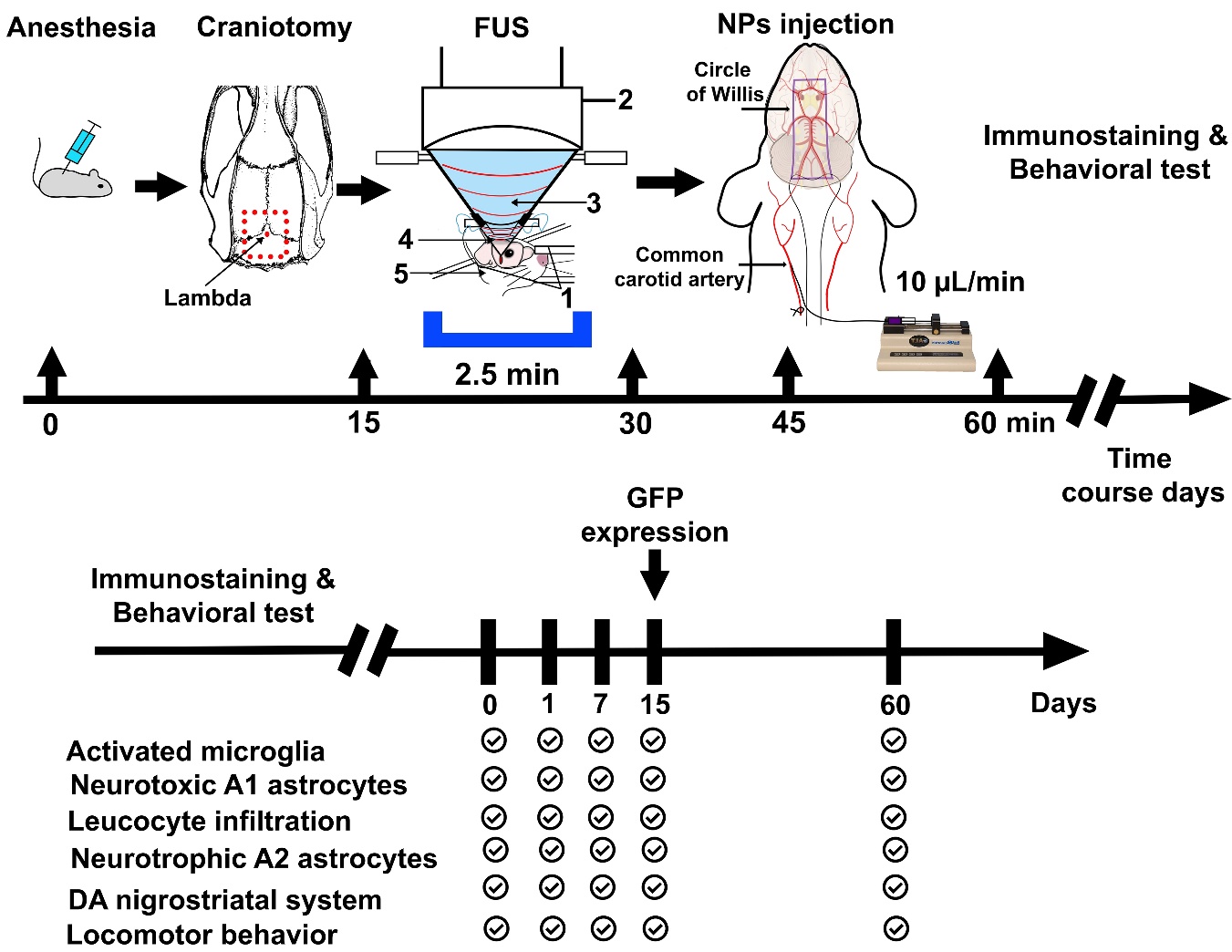
**

**Figure S1.** Experimental design for focus ultrasound (FUS) application, nanoparticle (NP)-mediated gene delivery via internal carotid artery, and neuroinflammation and neurodegeneration evaluation in the substantia nigra of Wistar rats. A similar protocol for FUS and biological parameter evaluations was followed for intravenous and intranasal gene delivery. FUS application diagram: 1. Stereotaxic, 2. transducer, 3. cone with water, 4. ultrasound transmission gel (Aquasoinc, Parker Laboratories; USA), 5. the rat. GFP = green fluorescent protein. DA = dopaminergic.


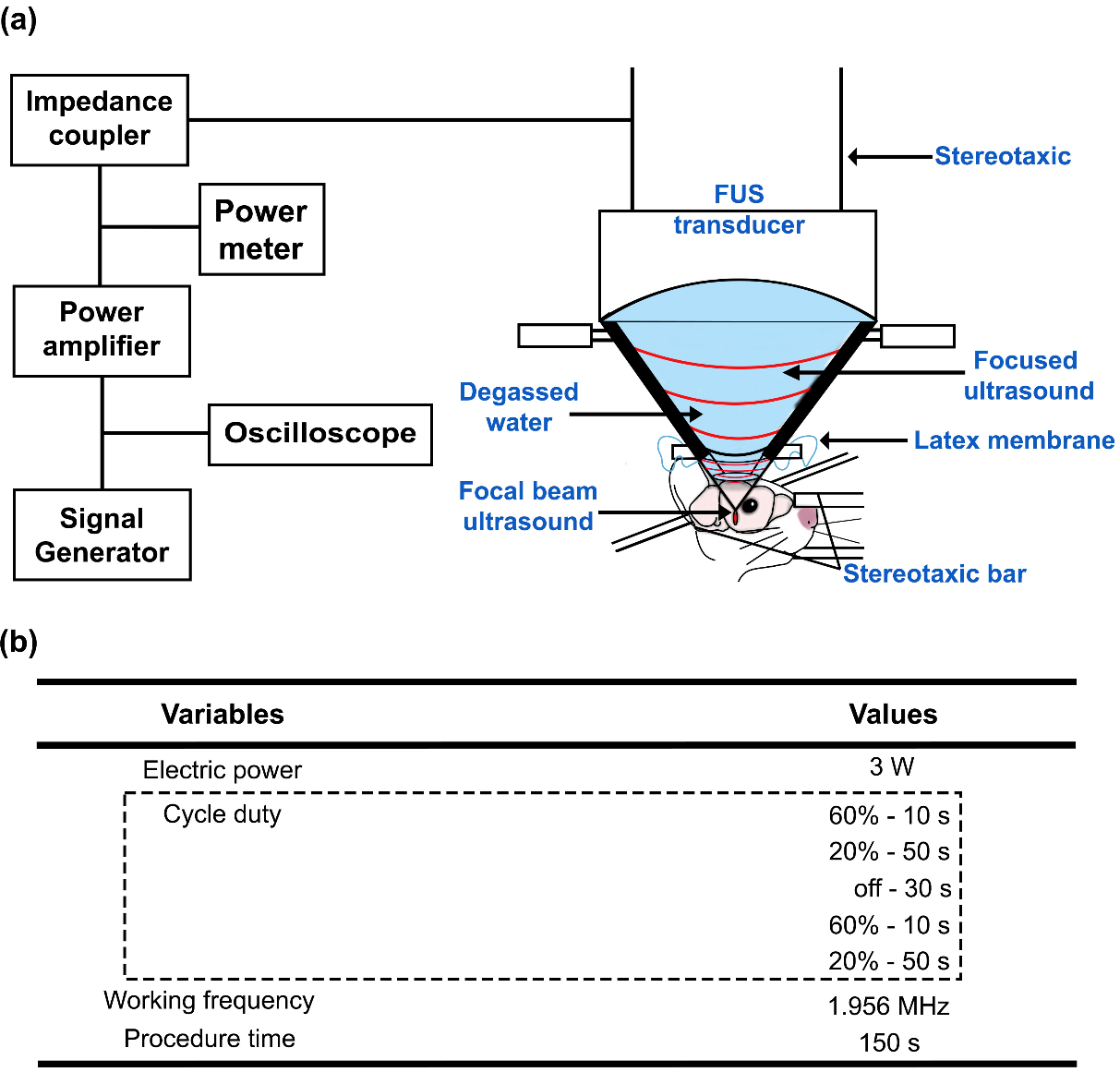


**Figure S2.** Array and parameters to open the brain-blood barrier transiently in the substantia nigra of adult male Wistar rats. **(a)** Focused ultrasound (FUS) setup, **(b)** Focused ultrasound parameters for transiently opening of the blood-brain barrier in the substantia nigra.

**Table S1.** Antibodies for double immunofluorescence

| **Epitope** | **Primary antibodies** | **Secondary antibodies** | **Objective** |
| --- | --- | --- | --- |
| TH | mouse monoclonal anti-TH | horse Texas red anti-mouse | Location of microglia in the substantia nigra |
| Iba1 | goat polyclonal anti-Iba1 | chicken Alexa Fluor 488 anti-goat |  |
| TH | rabbit polyclonal anti-TH | goat Texas red anti-rabbit | Astrocyte location in the substantia nigra |
| GFAP | mouse monoclonal anti-GFAP | chicken Alexa 488 anti-mouse |  |
| C3 | rabbit polyclonal anti-C3 | chicken Alexa 488 anti-rabbit | Activation of Astrocytes A1 |
| GFAP | mouse monoclonal anti-GFAP | horse Texas red anti-mouse |  |
| S100a10 | rabbit polyclonal anti-S100a10 | chicken Alexa 488 anti-rabbit | Activation of Astrocytes A2 |
| GFAP | mouse monoclonal anti-GFAP | horse Texas red anti-mouse |  |
| TH | rabbit polyclonal anti-TH | goat Texas red anti-rabbit | Leukocyte infiltration in the substantia nigra |
| CD45 | mouse monoclonal anti-CD45 | chicken Alexa 488 anti-mouse |  |
| TH | mouse monoclonal anti-TH | horse Texas red anti-mouse | GFP expression in dopaminergic neurons |
| GFP | rabbit polyclonal anti-GFP | chicken Alexa 488 anti-rabbit |  |
| **Iba1** (ionized calcium-binding adapter molecule 1), **TH** (tyrosine Hydroxylase), **GFAP** (glial fibrillary acidic protein), **C3** (complement component 3), **S100A10** (S100 calcium-binding protein A10), **CD45** (leukocyte common antigen), **GFP** (green fluorescent protein). | | | |

**Table S2.** Antibodies for immunohistochemistry staining

| **Epitope** | **Primary antibody** | **Secondary antibody** | **Objective** |
| --- | --- | --- | --- |
| TH | mouse monoclonal anti-TH | biotinylated horse anti-mouse IgG | Dopaminergic neurons |
| Iba1 | goat polyclonal anti-Iba1 | biotinylated horse anti-goat IgG | Activated microglia |
| GFAP | mouse monoclonal anti-GFAP | biotinylated horse anti-mouse IgG | Astrocytes |
| **Iba1** (ionized calcium-binding adapter molecule 1), **TH** (tyrosine hydroxylase), **GFAP** (glial fibrillary acidic protein) | | | |

**
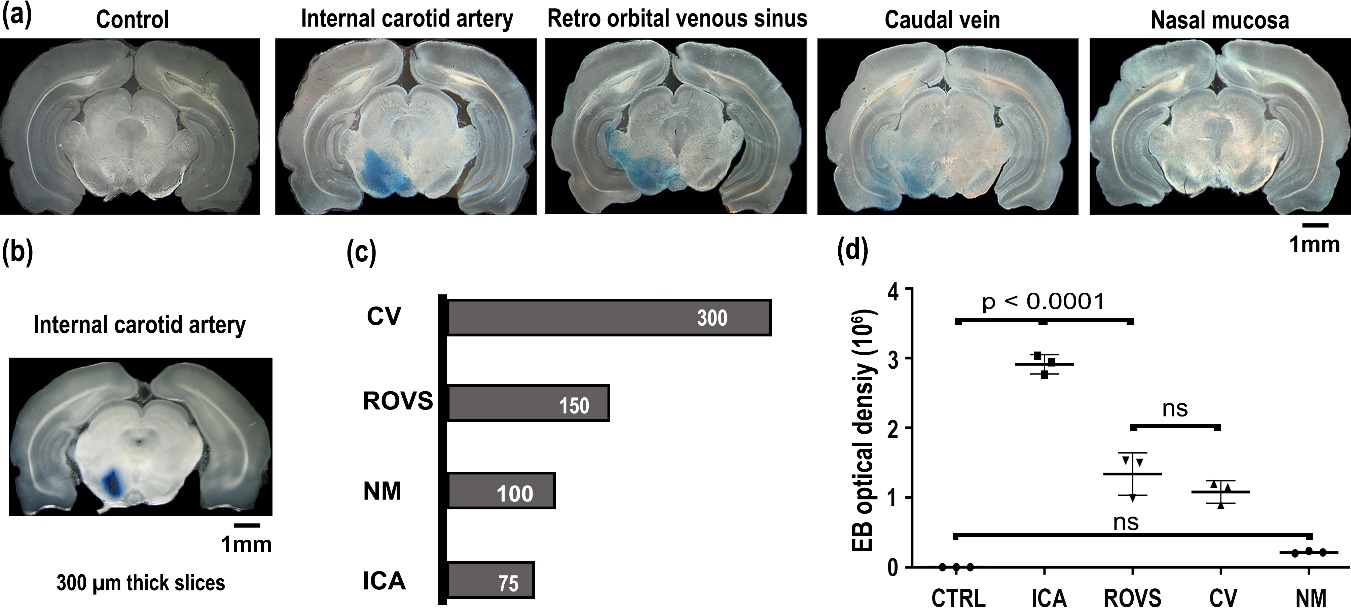
**

**Figure S3.** Focus ultrasound (FUS)-induced the blood-brain barrier opening in the substantia nigra allows Evans Blue (EB) dye extravasation after its systemic administration. Brains were dissected at 30 min after 2% EB dye administration. **(a)** Representative bright field micrographs of 30-µm thick coronal slices through the mesencephalon (Bregma – 6.04 mm, Paxinos & Watson Rat Atlas) of FUS-treated rats or without FUS (Control; CTRL) taken of fixed brains. **(b)** Representative micrographs of 300 µm-thick fresh mesencephalon taken 30 min after FUS. The value of the calibration bar is valid for all micrographs. **(c)** Graph indicating EB volumes injected into an internal carotid artery (**ICA**), retro-orbital venous sinus (**ROVS**), or caudal vein (**CV**) or deposited through a capillary tube on nasal mucosa (**NM**). **(d)** Graph of EB density using ImageJ software. The values are the mean ± SD from one anatomical level (n = 3 independent rats per experimental condition). One-way ANOVA and post hoc Tukey tests. ns = not significant.


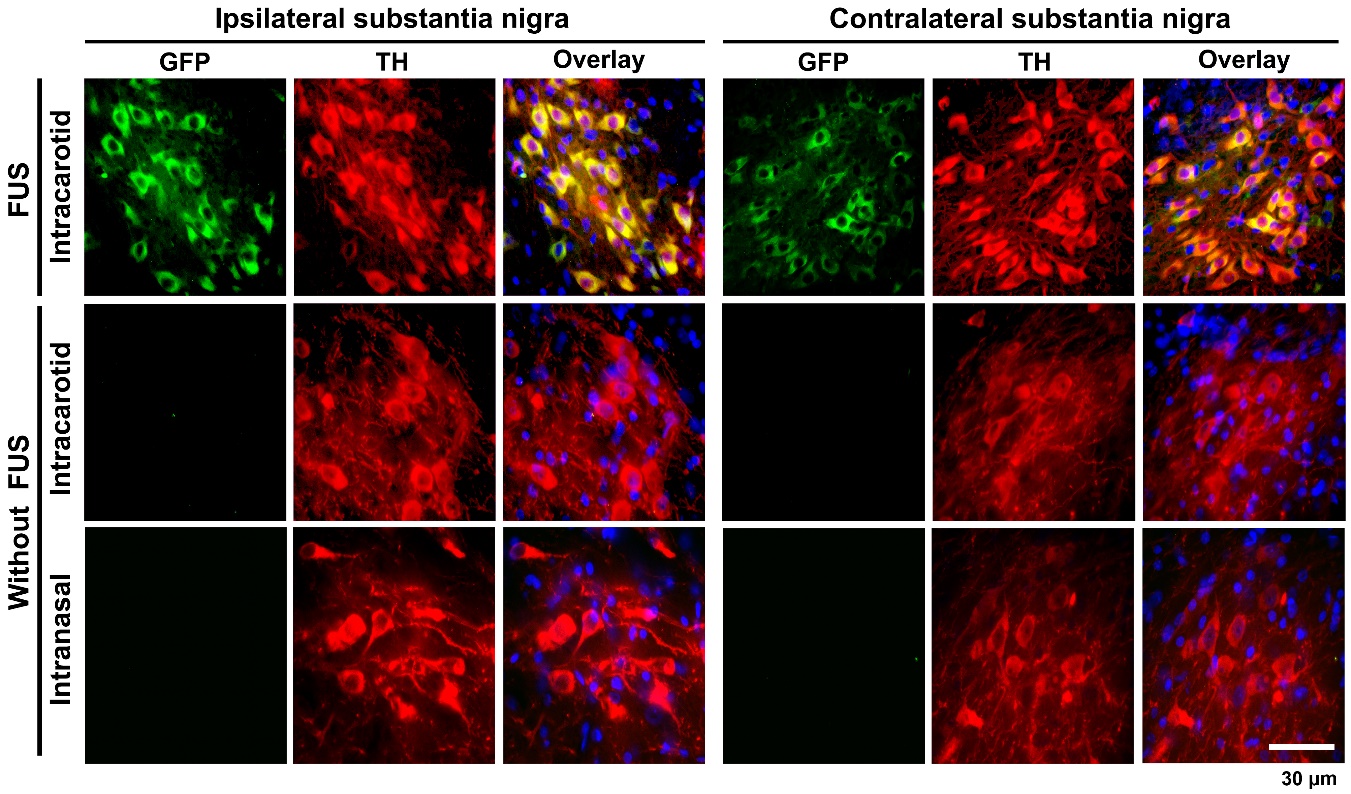


**Figure S4.** Figure S6. Absence of GFP fluorescence in the substantiae nigrae in animals administered with NTS-polyplex NPS via the intracarotid artery and nasal mucosa without FUS application. The micrographs are representative of three independent experiments made in triplicate. The micrographs of the top row correspond to Figure 2 top row (ICA) condition from rats transfected via an intracarotid artery (ICA) after FUS application used for comparison.

**
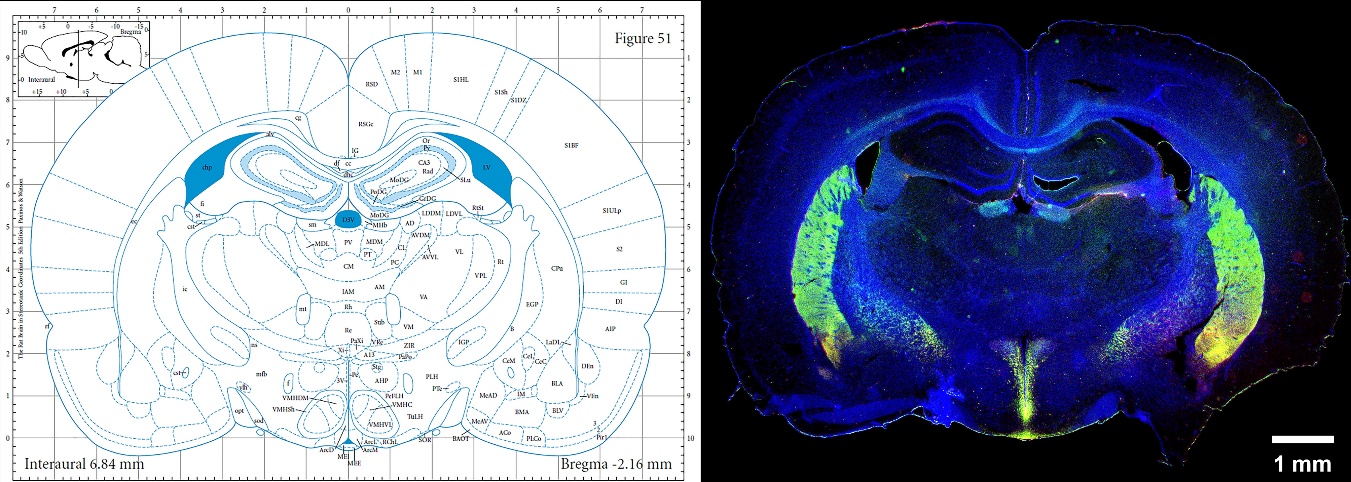
**

**Figure S5.** Identification of nuclei innervated with Green fluorescent protein GFP(+) axonal terminals. A comparison of the micrograph shown in Figure 3 with the illustration of Paxinos & Watson Rat Atlas at coordinates interaural 6.84 or Bregma -2.16 to locate the nuclei where the green fluorescent protein is present. Abbreviations of nuclei of interest: **CPu =** caudate putamen (striatum), **IGP** = internal globus pallidus. **EGP** = external globus pallidus. **3V** = Subventricular area of the third ventricle. GFP (green) immunoreactivity in Tyrosine hydroxylase (TH) (red) cells with nuclear Hoechst counterstaining (blue).

**
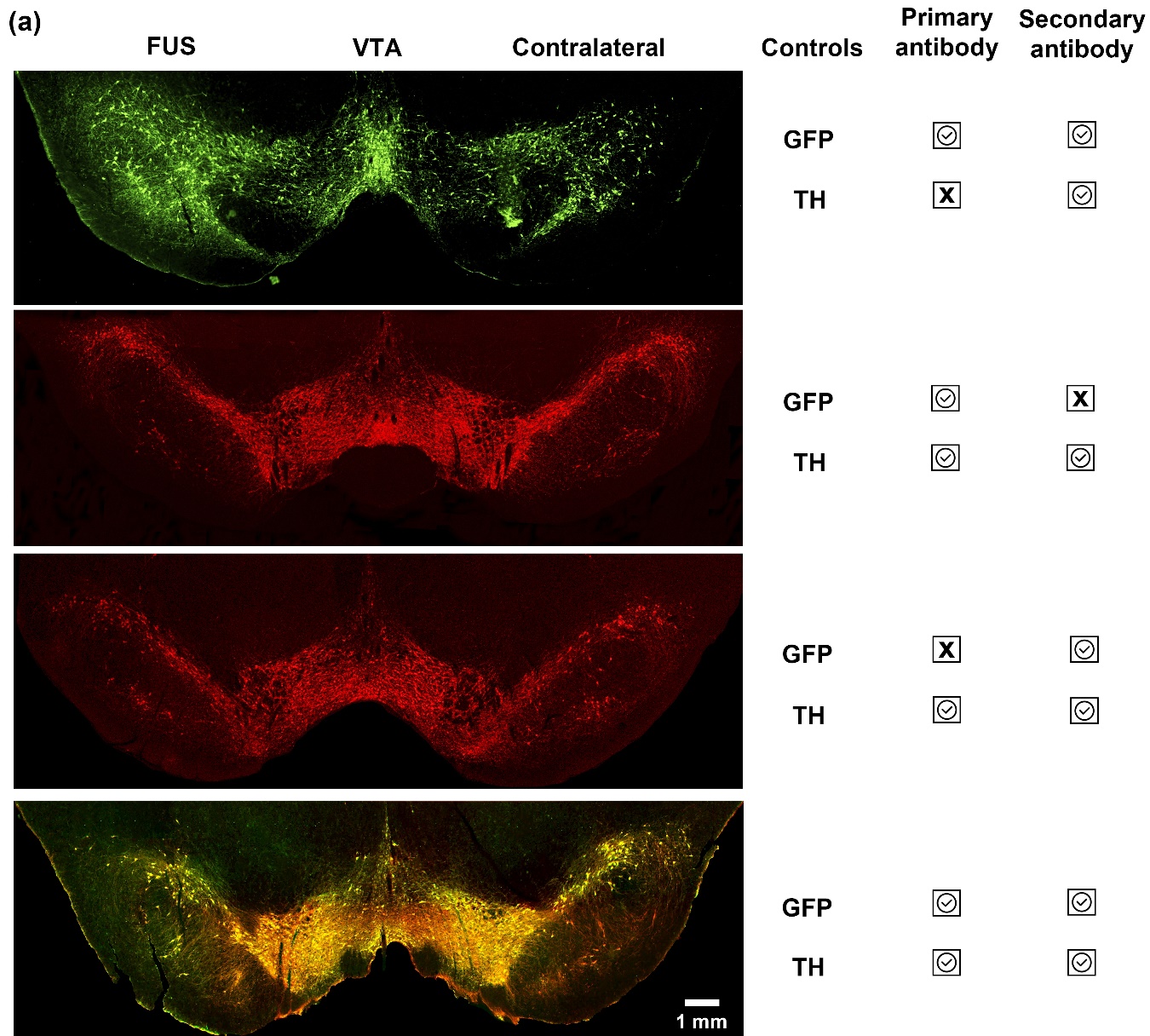
**

**Figure S6.** Controls of immunofluorescence staining after intracarotid transfection (n = 3 rats). Representative micrographs of the ventral mesencephalon were incubated with a suitable pair of antibodies or with only a primary or secondary antibody.
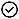
 = Use of primary or secondary antibody.
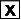
 = Omission of primary or secondary antibody. The value of the calibration bar is valid for the three micrographs. **VTA** = ventral tegmental area. **FUS** = Focus ultrasound, **GFP** = Green fluorescent protein immunoreactivity in **TH** = tyrosine hydroxylase (red) cells in the substantia nigra.


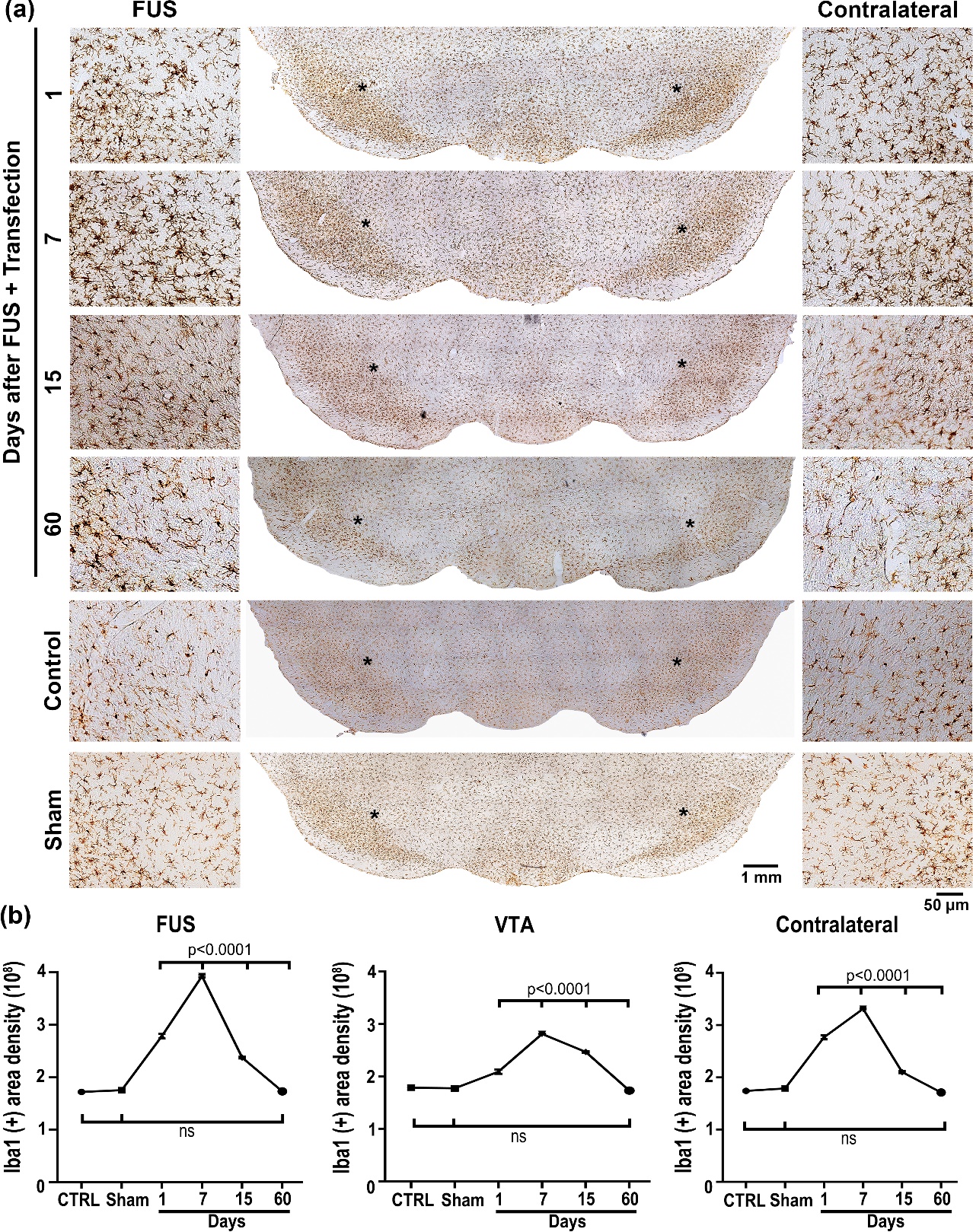


**Figure S7.** Reversible microglia activation after focus ultrasound (FUS) and transfection. **(a)** Representative micrographs of mesencephalon with ionized calcium-binding adaptor molecule 1 (Iba1) immunohistochemistry. The scale value is equal for the respective set of micrographs. **(b)** Scatter plots of Iba1(+) density measured on the whole area of the substantia nigra and ventral tegmental area (VTA) from micrographs of panel (a) using ImageJ software. The values are the mean ± SD from one anatomical level (n = 3 independent rats per experimental condition). One-way ANOVA and post hoc Tukey tests. ns = not significant.


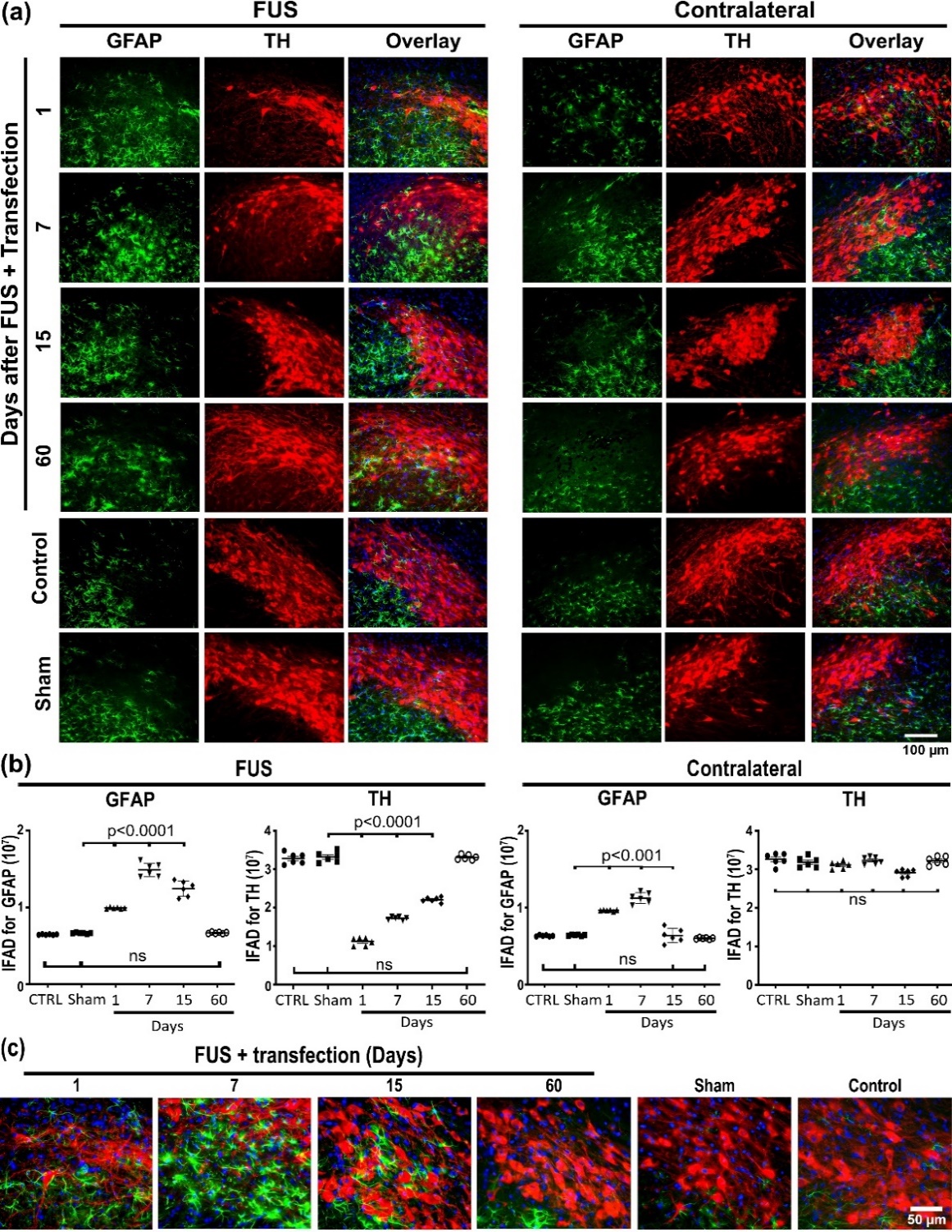


**Figure S8.** Focus ultrasound (FUS) causes transient astrogliosis in the substantia nigra. **(a)** Representative micrographs of the most injured site of the substantia nigra double immunostained with glial fibrillary acidic protein (GFAP) and tyrosine hydroxylase (TH) over time after FUS. The merged micrographs also include the Hoechst nuclear counterstaining. **(b)** GFAP and TH immunofluorescence area density (IFAD) measured from micrographs of panel A using ImageJ software. The values are the mean ± SD from three anatomical levels (n = 6 independent rats per experimental condition). One-way ANOVA and post hoc Tukey tests. ns = not significant. **(c)** Amplified micrographs show GFAP(+) cells with phenotypic changes according to their activation state [[55-57](#_ENREF_55)]. The scale value is equal for the respective set of micrographs. GFAP (green) immunoreactivity in TH (red) cells with nuclear Hoechst counterstaining (blue) in the substantia nigra over time.


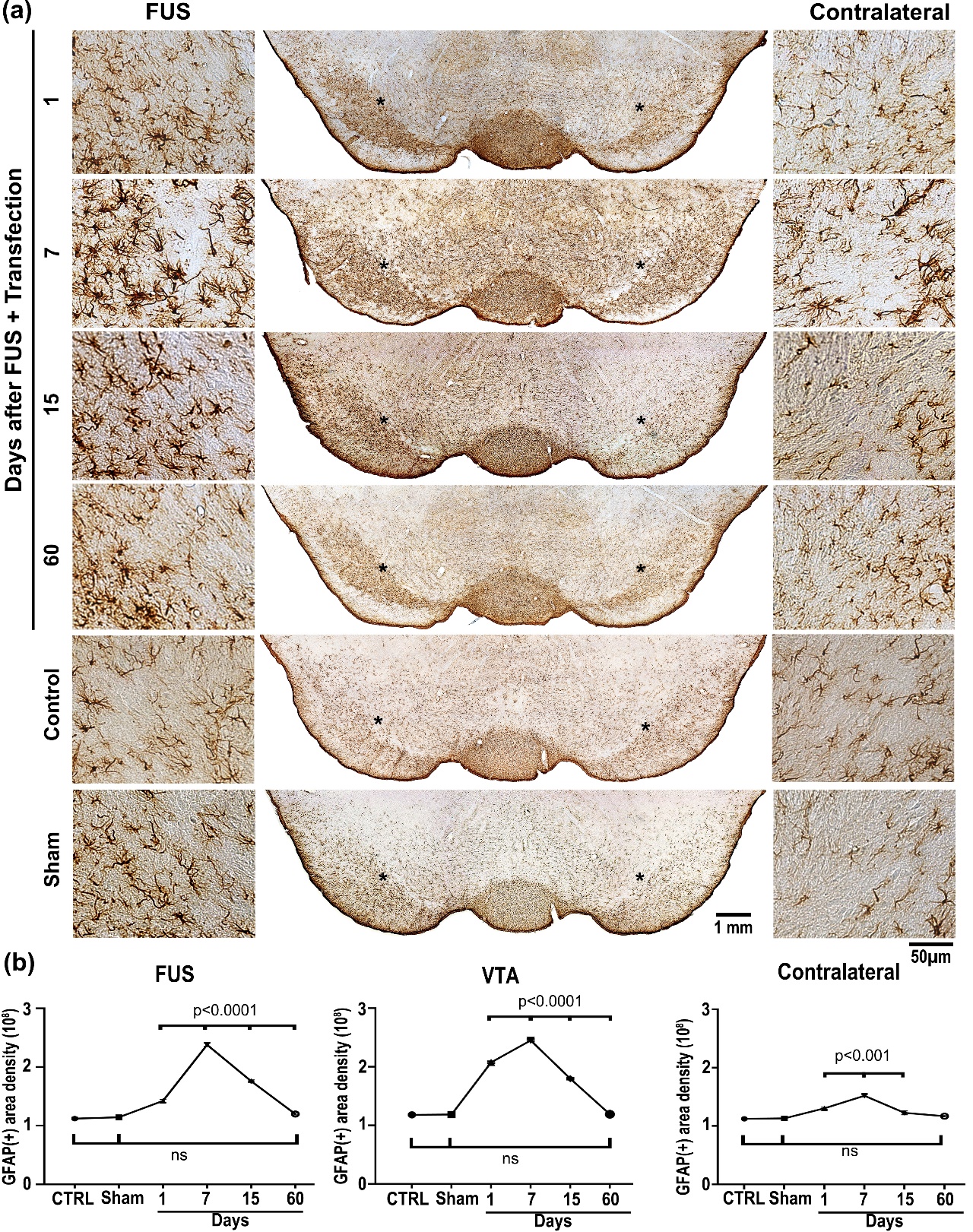


**Figure S9.** Reversible reactive astrogliosis after focus ultrasound (FUS) and transfection. Representative micrographs of mesencephalon with GFAP immunohistochemistry. **(a)** Representative micrographs of mesencephalon with glial fibrillary acidic protein (GFAP) immunohistochemistry. The scale value is equal for the respective set of micrographs. **(b)** Scatter plots of GFAP(+) density measured on the whole area of the substantia nigra and ventral tegmental area (VTA) from micrographs of panel A using ImageJ software. The values are the mean ± SD from one anatomical level (n = 6 independent rats per experimental condition). One-way ANOVA and post hoc Tukey tests. ns = not significant.
